# Long-read methylome analysis of *Oleidesulfovibrio alaskensis* G20 biofilm under copper stress

**DOI:** 10.1038/s41598-025-22029-8

**Published:** 2025-10-31

**Authors:** Payal Thakur, Ram Nageena Singh, Rajesh Kumar Sani

**Affiliations:** 1 Karen M. Swindler Department of Chemical and Biological Engineering, South Dakota Mines, Rapid City, SD USA; 2 2-Dimensional Materials for Biofilm Engineering, Science and Technology, South Dakota Mines, Rapid City, SD 57701 USA; 3 Data-Driven Material Discovery Center for Bioengineering Innovation, South Dakota Mines, Rapid City, 57701 SD USA

**Keywords:** Biofilm, Copper, DNA methylation, Oxford nanopore, Sulfate reducing bacteria, Genetics, Microbiology, Molecular biology

## Abstract

This study represents the first investigation of 5-methyl cytosine (5mC) DNA methylation patterns in sulfate-reducing bacterial (SRB) biofilms under copper (Cu) stress, utilizing Oxford Nanopore Technologies (ONT) sequencing. DNA methylation is a crucial epigenetic modification that is dynamic and regulates the signals to modulate molecular mechanisms across biological systems. The regulatory roles of DNA methylation in prokaryotic systems remain comparatively understudied than in eukaryotes. Bacteria are highly sensitive to environmental changes and therefore may utilize additional mechanisms like DNA methylation to combat the stresses. Our previous studies, utilizing microscopy and growth analyses, revealed that *Oleidesulfovibrio alaskensis* G20 (OA G20) biofilms responded to Cu stress. However, the DNA methylation patterns associated with this response remain unexplored, leaving a critical gap in our understanding of the epigenetic mechanisms regulating OA G20 biofilms under Cu stress. This study aims to address this knowledge gap by identifying 5mC DNA methylation in biofilms of OA G20 under Cu stress. To achieve our goal OA G20 biofilms cultivated under 30 µM-Cu ion stress along with control and sequenced through ONT sequencing. DNA methylation analysis was performed using the MicrobeMod pipeline identifying three methylated motifs: TCCG, CCCGCCCG, and CGGGAT in control (0 µM-Cu). TCCG was identified as the predominant methylated motif, with analysis revealing 78,022 genomic positions in the control condition. Of these, 61.7% exhibited 5mC modifications, 33.9% remained unmodified, and 4.4% showed uncharacterized modifications. In contrast, the 30 µM-Cu biofilm showed methylation in only two motifs, TCCG and GCANCTGCGS. Analysis of TCCG revealed 63,315 genomic positions, with 62.7% (39,706 sites) showing methylation and 33.2% (20,990 sites) remaining unmethylated. A total of 1418 common methylated positions were identified for both conditions and there were 341 and 424 genomic positions identified for motif TCCG above 75% methylation in the 0 µM and 30 µM-Cu biofilm samples, respectively. Differential methylation analysis revealed significant variations in methylation patterns across several key genes of crucial molecular pathways, important for biofilm formation, including ATP-Binding Cassettes (ABC) transporters, phosphohydrolase, flagellar biosynthesis, chemotaxis, cobalamin synthase, histidine kinase, and uncharacterized proteins.

## Introduction

Genomes are constructed by universal building blocks known as nitrogenous bases A, T, C and G in both prokaryotes and eukaryotes. The arrangement of these nitrogenous bases holds genetic information and directs the cell to perform different functions through codons and replicons. To combat external influence, the genome performs dynamic (reversible) changes without altering the nucleotide arrangement known as DNA modifications. DNA modification is one of the gene expression regulation mechanisms other than noncoding RNAs^1^. The modification in these DNA bases does not change the underlying sequence instead carries additional information^2,3^. In bacteria, DNA modifications are catalyzed by methyltransferases, which transfer methyl groups to adenine and cytosine bases, facilitating the formation of N6-methyladenine (6mA), N4-methylcytosine (4mC), or C5-methylcytosine (5mC)^3,4^. DNA methyltransferase (DNMTs) catalyzes the transfer of a methyl group to these sites from a donor molecule, like S-adenosyl-L-methionine (SAM)^5,6^. Most prokaryotic DNMTs are well-studied for their role in the restriction-modification (R-M) system, which protects the chromosomal DNA from cognate restriction enzymes.

DNA methylation provides defense against bacteriophage, where the restriction enzyme digests the unmethylated phage DNA^7–9^. In addition to this role, DNA methylation has been studied in the context of other biological functions in several bacteria such as *E. coli*^10,11^, *S. enterica*^12,13^, *Caulobactor*^14,15^, and other genera where methylated bases were involved in cell cycle^16,17^, antibiotic resistance^18,19^, mismatch repair^20,21^, cellular phenotypes^10,22^ and biofilm formation^23,24^ in response to environmental conditions. When methylated bases regulate important cellular processes outside of R-M systems, the enzymes that create these modifications are classified as orphan or solitary DNMTs^18,25,26^.

With the development of sequencing technologies like single-molecule real-time (SMRT) sequencing, Oxford Nanopore Technology (ONT), and bisulfite sequencing, some research groups have sought to elucidate the role of DNA methylation in prokaryotes^27–30^. A few examples include; DNA methylation patterns corresponding to the growth state of *E. coli*, eliminating the need for any other transcriptomic or proteomics analysis^10^; DNA methylation controls cell motility and biofilm formation in *B. cenocepacia*^23^; and regulation of *sugE* gene which mediates antimicrobial resistance in *E. coli*^11^.

The role of 5mC has been extensively studied in eukaryotic systems for its role in regulating gene expression, cellular differentiation, and impact on diseases like cancer whereas in prokaryotes, 6mA and 4mC have been mainly linked with gene expression regulation^4,18,28^. However, the role of 5mC in bacterial gene expression remains largely unknown.

*Oleidesulfovibrio alaskensis* G20 (OA G20) is a Gram-negative sulfate-reducing bacterium (SRB) with approximately 58% GC content^31^. The industrial importance of SRB is due to their dual role in the biocorrosion of metals^32–36^ and bioremediation of heavy metal ions^37–39^, highlighting these bacteria as important targets for scientific investigation. For decades copper (Cu) has been used as an anti-microbial agent^40–42^, however, SRB has developed several strategies to combat Cu stress. One of the key strategies adopted by SRB to overcome heavy metal stress is the development of biofilms, which is the aggregation of cells encapsulated in a self-produced extracellular matrix^43–46^. Our previous work on OA G20 demonstrated that OA G20 employs several mechanisms to overcome Cu stress which includes biofilm formation, overproduction of extracellular matrix, regulation of genes involved in stress response, cell motility, efflux pumps, quorum-sensing, and chemotaxis^47,48^. Albeit, transcriptomic response to Cu stress in OA G20 has previously been reported^48^, no link between 5mC DNA methylation and OA G20 biofilm formations and its response to Cu stress has yet been established.

Therefore, to probe the potential role of DNA methylation in SRB, here we present the first study of 5mC methylation patterns in OA G20. The main aim of the current study is to study profile changes in DNA methylation patterns when OA G20 biofilms are exposed to Cu ion stress. These DNA methylation patterns were captured by performing long-read DNA sequencing using ONT sequencing. To process the sequenced data, the MicrobeMod pipeline^49^ was utilized to analyze methylation levels in OA G20 biofilms, comparing biofilms exposed to 30 µM-Cu stress to control (0 µM-Cu). Our analysis revealed that methylation levels vary across the genome and show distinct differences in 30 µM-Cu (CuB) vs. 0 µM-Cu (CB) biofilm samples. The analysis identified regions where percentage methylation (PM) changes under Cu stress conditions. These results provide the first quantitative map of 5mC methylation changes in OA G20 during Cu exposure, establishing the baseline for future studies investigating the potential role of DNA methylation in bacterial stress responses particularly in SRB.

## Materials and methods

### Bacterial growth

OA G20 seed cultures were grown as previously described^47,48^. A lactate-C medium was sterilized as the growth media by autoclaving (15 min, 15psi, 121℃). Serum bottles containing 100 ml of lactate-C medium were made anoxic by sparging filter-sterilized ultrapure nitrogen for 20 min at 10 psi. The media was then inoculated with 2 ml of OA G20’s frozen glycerol stock (40% v/v) inside an anaerobic chamber (COY Lab Products, Grass Lake, MI, USA). The cultures were grown to mid-log phase at 30℃, 125 rpm or until they reached an OD600 between 0.13 and 0.15. The active culture was sparged with filter-sterilized ultrapure nitrogen for one hour inside an exhaust hood to remove hydrogen sulfide (H_2_S) and then the cells were pelleted by centrifugation at 10,000×g for 10 min. The supernatant was removed followed by washing the cell pellet with anaerobic PBS (50 mM, pH 7.2) by centrifuging for 3 min at 10,000×g. After washing, the cells were resuspended in anoxic PBS and a 5% v/v inoculum was used for biofilm experiments. All the steps including inoculum transfer and cell washing were performed inside the anaerobic chamber.

### Copper treatment and biofilm formation

A filter-sterilized 0.05 M CuCl_2_ stock solution was prepared to induce OA G20’s biofilm formation under Cu stress as previously described^47^. The current study utilized glass slides submerged in serum bottles containing 100 ml of anoxic lactate C medium to allow the formation of biofilm. The serum bottles were then supplemented with 0.05 M CuCl_2_ to achieve the final concentration of 30 µM and the serum bottles without the supplementation of CuCl_2_ were used as control (0 µM). The active seed culture (5% v/v), prepared during the logarithmic phase of bacterial growth, was used to inoculate all serum bottles, including the controls, inside the anaerobic chamber. Biofilms were allowed to grow for a total of seven days at 30 ℃, 25 rpm followed by measurement of Cu ion concentrations and DNA extraction for further analysis.

### Genomic DNA extraction and quantification

For DNA methylation analysis, OA G20 biofilms were harvested on day 5 as previously described^47^. Next, the genomic DNA was extracted using MasterPure™ Complete DNA Purification Kit protocol (Lucigen, Radnor, PA, USA). DNA quality assessment and quantification was performed using Quant-iT dsDNA Broad-Range (BR) assay kit with a Qubit 4.0 fluorometer (Thermo Fisher Scientific, Waltham, MA, USA). The DNA samples were stored at -20℃ until library preparation and sequencing.

### Library Preparation and nanopore sequencing

**Fig. 1:**
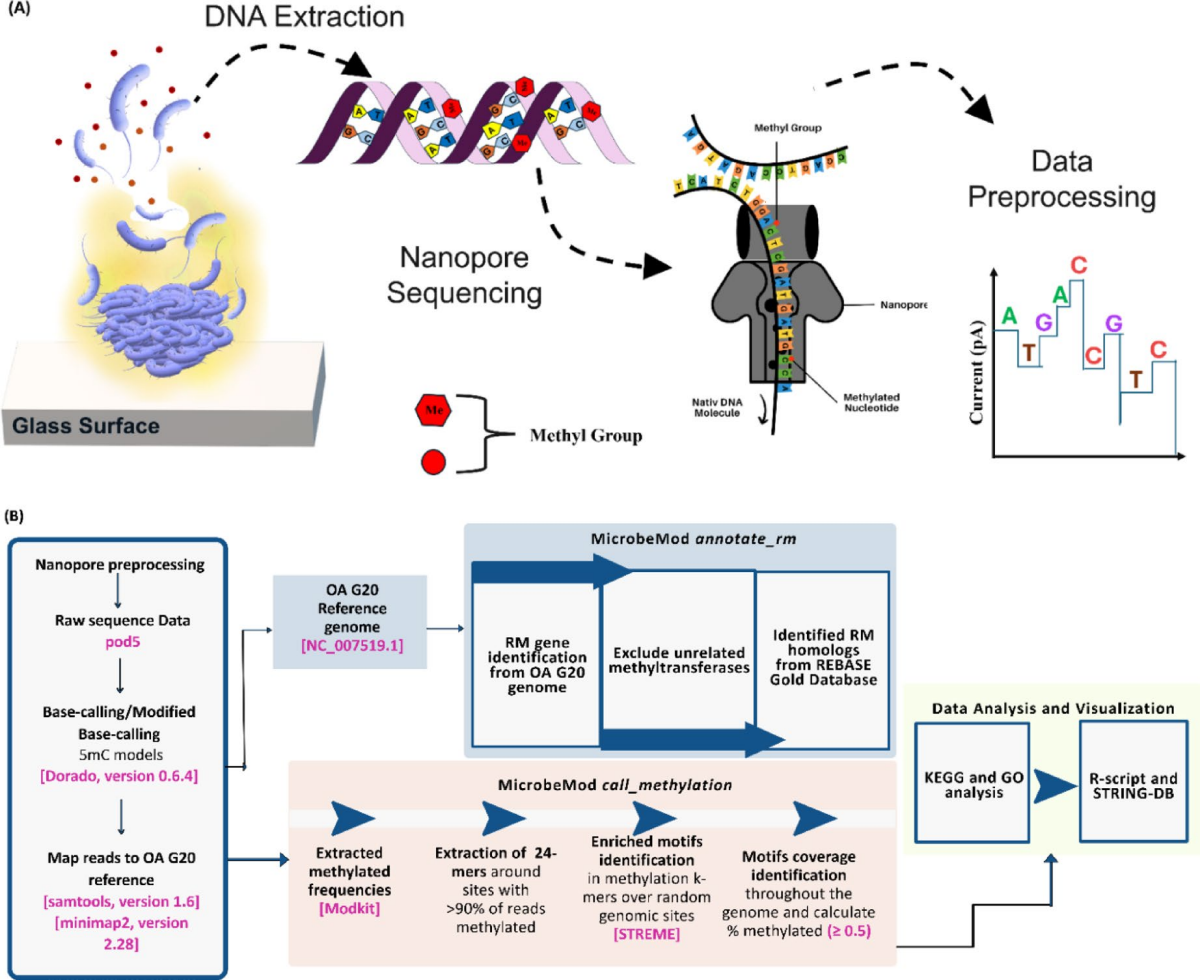
Conceptual overview of DNA methylation analysis pipeline. (**A**) The biofilm samples were harvested and sequenced using an ONT sequencing device. (**B**) The raw data was processed using two separate pipelines integrated into the MicrobeMod toolkit. The data analysis was done using GO functional and KEGG pathway analysis followed by data visualization using R-scripts and STRING-DB.

To determine the methylome of OA G20 biofilms, DNA samples were analyzed using ONT sequencing. The ONT sequencing libraries were prepared using the SQK-LSK109 ligation sequencing kit with barcode expansion EXP-NBD104 kit, following the manufacturer’s instructions. The sequencing library was quantified using Quant-iT 1X HS dsDNA assay kit on Qubit 4.0 fluorometer (Thermo Fisher Scientific, Waltham, MA, USA). After the library preparation, ONT sequencing was performed using a MinION Mk1B device on R9.4.1 flow cell. The sequencing and raw data collection was performed on MinKNOW software version 24.02.16. (Fig. 1A).

### Identification of restriction-modification (R-M) enzymes

R-M systems were annotated using ‘*annotate_rm*’ pipeline from MicrobeMod (version 1.0.4). The OA G20 reference genome (NC_007519.1) was utilized as an input for the identification of methyltransferases and restriction enzymes belonging to R-M systems. The ‘*annotate_rm*’ pipeline utilized a built-in REBASE Gold database^50^ which consists of detailed information on experimentally validated sequence motifs, DNMTs, and R-M systems^49,50^. The analytical approach for DNA methylation data processing is outlined in Fig. 1B, which presents the conceptual overview of the pipeline followed in our current study.

### DNA methylation analyses

#### Preprocessing of raw sequence data and base calling

After the completion of the sequencing, the raw sequenced data (.fast5) was converted to pod5 files using pod5 (version 0.3.23). The pod5 files were further processed for basecalling followed by demultiplexing for each sample according to the barcodes using Dorado. To identify the methylated motifs, base calling was done using Dorado (version 0.6.4) with a modified basecalling model (dna_r9.4.1_e8_hac@v3.3), which contains an unmapped BAM file including modified base information.

#### Mapping based called reads with reference

After modified basecalling, the basecalled reads were mapped to the OA G20 reference genome using samtools (version 1.6) and minimap2 (version 2.28)^51^. It is important to note that once the mapped result files must contain MM tags, which encode the type and position of modified bases such as methylated cytosines, while the ML tags store the confidence scores for each reported modification to ensure accurate data processing^52^.

#### Detection of 5mC methylation using microbemod

MicrobeMod pipeline was used to detect 5mC DNA methylation following the workflow (Fig. 1) as previously described by^49^ with a few modifications in the default parameters. The mapped BAM files were processed using the MicrobeMod call_methylation command with parameters; min_strand_coverage = 0.5, methylation_confidence_threshold = 0.5, pecent_methylation_cutoff = 0.5, and percent_cutoff_streme = 0.5.

### Data visualization and analysis

The data visualization was carried out using web-based tools and custom R programming scripts (Supplementary File S1). To gain deeper insights into the data, pathway enrichment analysis using the STRING database along with Gene Ontology (GO) functional analysis was performed. Finally, these results and findings were integrated with information from UniProtKB^53^, the Kyoto Encyclopedia of Genes and Genomes (KEGG)^49,54^ and existing scientific literature. The experiments were performed in biological duplicates. However, comprehensive statistical analysis between biological replicates could not be performed due to technical limitations of the MicrobeMod pipeline, as it required single-sample input for quantitative analyses. Therefore, the data analysis focused on methylated positions that were common across both biological replicates within each condition, ensuring reproducibility of findings.

## Results

### Effect of copper ions on OA G20

The effect of Cu ions on the planktonic growth and biofilm formation of OA G20 has been previously explored by our group^47,48,55^. Our analysis revealed that Cu ions negatively affect the growth of OA G20 in its planktonic state^48^. On the contrary, when OA G20 was allowed to grow biofilm when exposed to variable Cu concentrations of 5 µM, 15 µM, and 30 µM, it showed an enhanced biofilm formation compared to the control (0 µM-Cu)^47^. Following our previous study on OA G20 biofilm formation under Cu stress, we expanded our investigation to include Cu ion measurements and DNA methylation analysis to gain a better understanding of OA G20’s response to metal ion stress. The experimental conditions chosen for this study were the biofilm samples from the control, which were then compared to cells exposed to 30 µM-Cu ions. The maximum amount of physiological and quantitative changes in biofilms were observed in samples exposed to 30 µM-Cu, therefore, this concentration was utilized for comparison purposes. To understand the effect of Cu on the OA G20 biofilms, ONT sequencing was employed to elucidate changes in DNA methylation patterns triggered by metal ion stress.

### DNA sequencing results and quality assessment

The purified genomic DNA from both samples was quantified using Qubit fluorometry, yielding concentrations of 25 ng/µL for CB and 47 ng/µL for CuB, respectively. The DNA library was then constructed using standard protocols, and the pooled library was quantified at 19.7 ng/µL. A total of 11 µL (~ 90 fmol) of purified library was loaded onto a MinION flow cell. The sequencing run produced a total of 326,413,589 bases (326 Mb) in 43,450 reads where;14,819 reads (188,173,791 bp) were generated for CB, and 28,631 reads (138,239,798 bp) for CuB samples (Supplementary Figure S1 and File S2). The quality assessment of raw sequence reads was conducted using high-accuracy basecalling in Dorado v0.6.4. Sequencing data analysis revealed GC contents of 56.62% for CB and 56.30% for CuB, with corresponding mean read lengths of 12,574 bp and 4,905 bp, respectively. The sequenced reads exhibited uniform GC composition without any ambiguous base calls (N). Read alignment analysis demonstrated mapping efficiencies of 98.71% for CB and 98.53% for CuB.

### Identification of methylated motifs

**Fig. 2:**
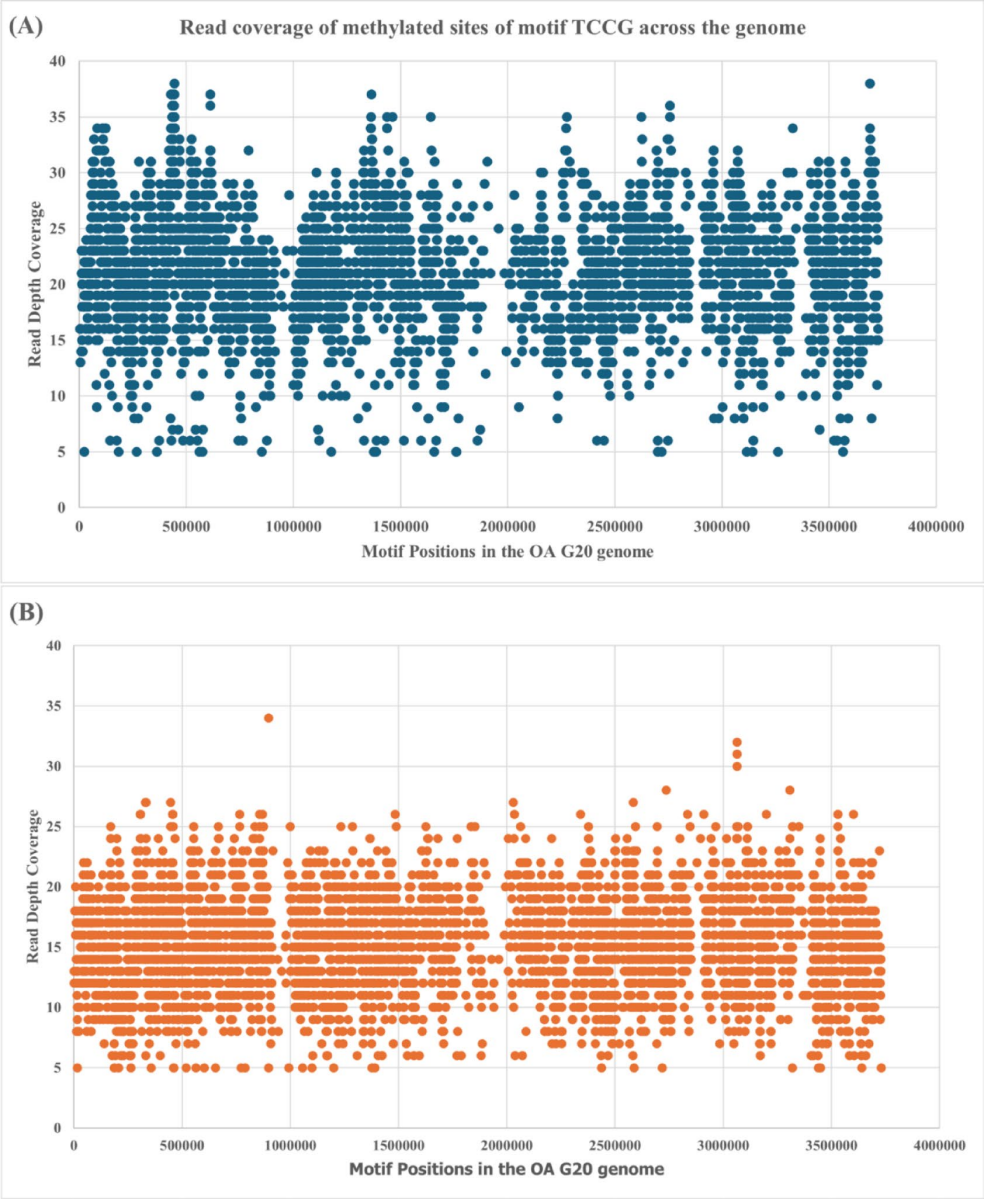
Distribution graphs of sequencing depth at TCCG motif of OA G20 genome. (**A**) Control biofilm (0 µM-Cu) and (**B**) 30 µM-Cu biofilm. The figures show the read depth coverage across genomically distributed TCCG methylation sites, with the majority of methylated sites exhibiting sequencing depths ranging from 15–30 high-quality consensus reads.

To check for the horizontal and vertical coverage of reads of the OA G20 genome, de novo genome assembler Flye 2.9^56^ was used, which resulted in 60x depth coverage (analysis not mentioned). Furthermore, to analyzed the depth of the TCCG motif, read depth analysis was performed which revealed that each identified motif base was supported by a minimum of 5 consensus reads, with vertical coverage reaching up to 38 reads for sample CB (Fig. 2A) and ranging from 5 to 34 reads for sample CuB (Fig. 2B). This comprehensive read depth coverage, combined with high sequencing accuracy and stringent methylation thresholds, ensures reliable identification of methylated bases across the genome while accounting for potential sequencing errors and technical variability.

In bacteria, DNA methylation is analyzed using the motif recognition approach^29^. Initial efforts focused on determining which DNMTs were originally present in the reference genome of OA G20. The use of the ‘*annotate_rm*’ pipeline from MicrobeMod, resulted in the identification of a total of three R-M enzymes: Type I, II, and III (Table 1). Among the R-M enzymes, Type_II_MTases were identified as the sole mediators of 5mC methylation at the GGATC motif. To further understand the difference in 5mC methylation patterns in OA G20 under stress, we conducted ONT sequencing (R9.4.1 MinIon flow cells) of samples collected from biofilms exposed to 30 µM-Cu ion concentrations and then compared them to the control (0 µM-Cu). For all the samples, 60x coverage was achieved on an average. Identification of modified genomic sites was done using MicrobeMod pipeline. The raw reads from ONT were aligned to the reference genome of OA G20. The *call_methylation* pipeline from MicrobeMod identified methylated sites which were further passed to STREME, resulting in significantly enriched methylated motifs with an e-value of 0.1^49^. The Supplementary File S3 shows the final output from the *call_methylation* pipeline, it contains details for all the methylated motifs found in our samples. For each motif, Table 1 includes their methylation type, its frequency and the position of methylation.

**Table 1:** DNMTs identified by MicrobeMod pipeline for reference genome of OA G20.

System Type	E-value	Homolog methylation	Homolog motif	REBASE homolog
RM_Type_III	8.5e^− 123^	6 mA	NA	M.DdeGORF1743P^1^
RM_Type_III	1.2e^− 61^	6 mA	NA	M.DdeGORF1743P^1^
RM_Type_I	1.4e^− 244^	6 mA	NA	M.DdeGORF1861P^1^
RM_Type_I	2.6e^− 182^	6 mA	NA	M.DdeGORF2498P^1^
RM_Type_I	3e^− 255^	6 mA	NA	M.DdeGORF2868P^1^
RM_Type_I	1.1e^− 177^	6 mA	NA	M1.DdeGORF3427P^1^
RM_Type_I	7.9e^− 159^	6 mA	NA	M2.Asp64203ORF9495P^1^
RM_Type_II	5e^− 21^	5mC	GGATC	M.DdeGORF922P^1^
RM_Type_II	5.5e^− 36^	5mC	GGATC	M.DdeGORF1904P^1^

^1^ Putative DNMTs which are not experimentally validated in REBASE Gold database.

**Fig. 3:**
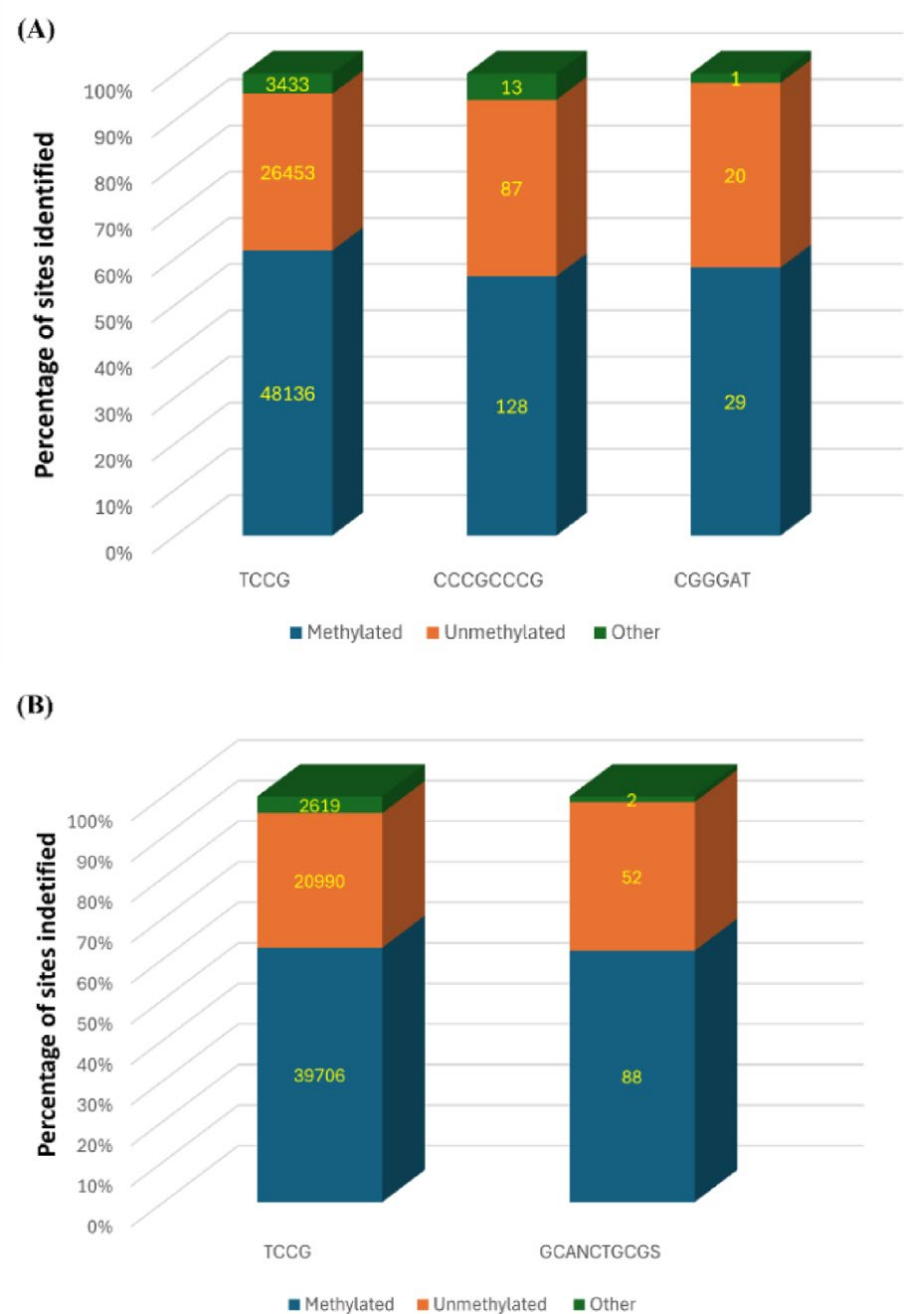
Distribution of methylated and unmethylated sites on identified motifs. (**A**) Control biofilm (0 µM-Cu) and (**B**) 30 µM-Cu biofilm. The chart depicts the distribution of DNA modification status: methylated positions (blue), unmethylated positions (orange), and modified sites with uncharacterized modification type (green).

From the output table of *call_methylation* pipeline, we determined that on average, OA G20 contained two methylation motifs for 5mC DNA methylation. In control (0 µM-Cu), three motifs; TCCG, CCCGCCCG, and CGGGAT were found to be methylated. Among the identified methylation motifs, the TCCG was identified as the most methylated motif. Figure 3 shows a total of 78,022 genomic positions were found in the control, demonstrating significant methylation activity. Of these positions, 48,136 sites (61.7%) exhibited 5mC modifications, while 26,453 sites (33.9%) remained unmodified. The remaining 3,433 sites (4.4%) showed evidence of modification, although the specific nature of these modifications was not characterized in our current experiment. In comparison to the TCCG motif, CCCGCCCG and CGGGAT showed moderate levels of methylation.

For biofilm samples containing 30 µM-Cu ion concentration, TCCG and GCANCTGCGS motifs were found to be methylated. Under 30 µM-Cu treatment conditions, analysis of the TCCG motif identified 63,315 genomic positions. Of these, 62.7% (39,706 sites) exhibited methylation modifications, while 33.2% (20,990 sites) remained unmodified (Fig. 3). Compared to the TCCG motif, GCANCTGCGS displayed intermediate methylation, where N represents any nucleotide (A, T, G, C) and S stands for the presence of either G or C^57^. Therefore, this study centered on analyzing methylation patterns only across TCCG motifs for two key reasons. First, TCCG motifs showed significant methylation in both the experimental conditions (0 µM-Cu and 30 µM-Cu), making them suitable for comparative analysis. Second, TCCG represented a common motif present across both conditions, providing a consistent baseline for comparison. By focusing on TCCG motif, we established a comparative framework to evaluate the effects of Cu ions on methylation patterns in OA G20 biofilms between test and control conditions. Subsequently, OA G20 promoter regions were predicted using *bprom* algorithm (SoftBerry). We observed that no genomic positions were methylated in the promoter region when compared to the raw methylation data (Supplementary File S4).

The *annotation_rm* pipeline revealed that Type_II_MTases mediated 5mC methylation at the GGATC motif. Interestingly, under the current experimental conditions, 5mC methylation was not detected at GGATC sequence motifs, instead it was observed at alternative motifs like TCCG, CGGGAT. The detailed analysis of alternative motif-methylation patterns in OA G20 is beyond the scope of our current study. While no DNMTs in the REBASE database have been experimentally confirmed to target TCCG and other above-mentioned motifs, a possible explanation for the introduction of new methylation patterns at these motifs can be attributed to the presence of uncharacterized or orphan DNMTs^29,58^ or changes in the environmental conditions^59^.

### Determination of global methylation patterns

**Fig. 4:**
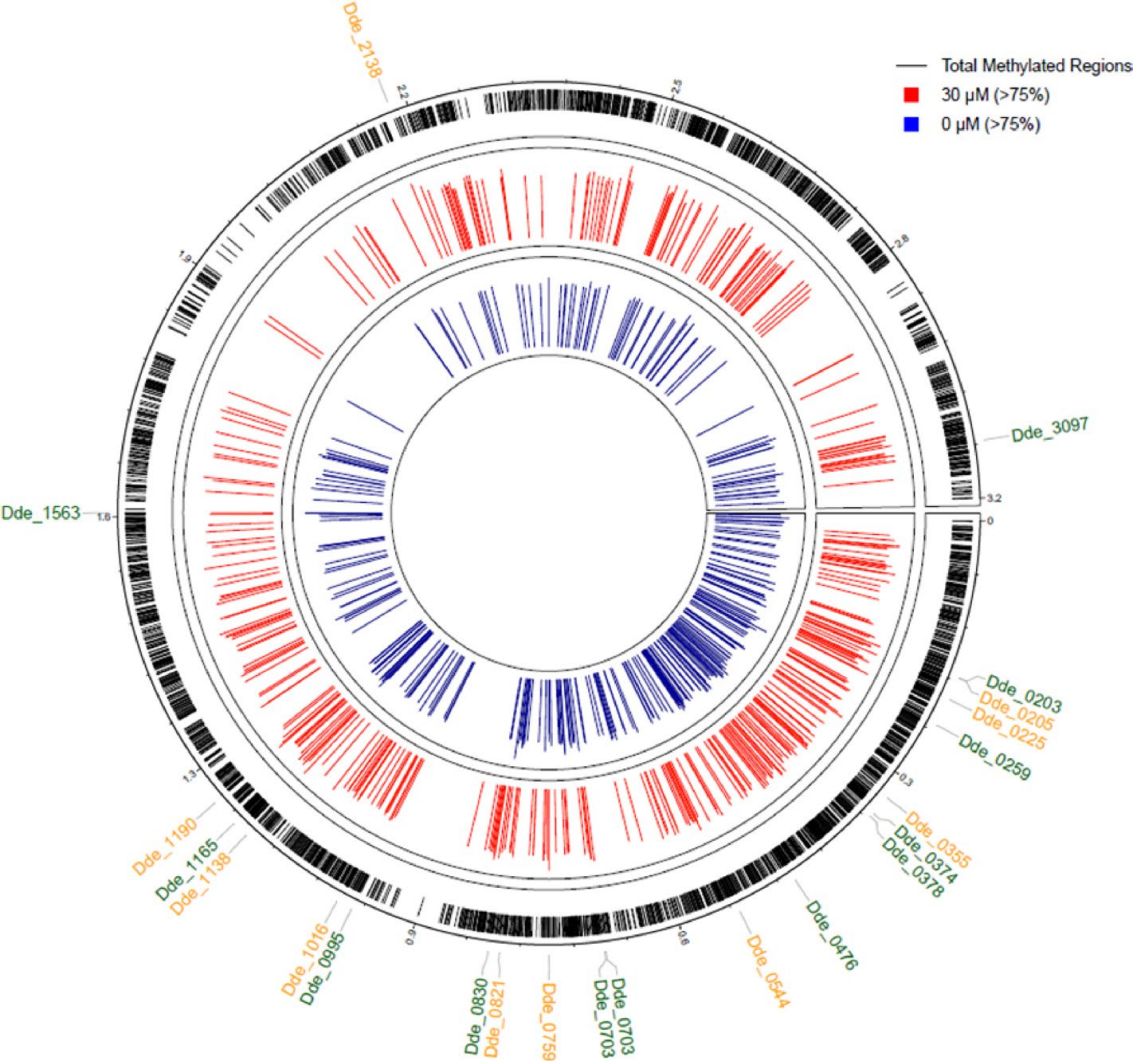
Distribution of TCCG motif methylation across the OA G20 genome. The figure illustrates the genomic locations of methylated TCCG motifs, with black tick marks representing the total methylated positions throughout the genome. Positions exhibiting methylation levels exceeding 75% are highlighted in red for Cu-stressed conditions (30 µM-Cu) and blue for control conditions (0 µM-Cu). Gene identifiers present under both experimental conditions are denoted with green labels (0 µM) and orange labels (30 µM).

To examine the variations in 5mC DNA-methylation between control and stress conditions, methylated positions at the TCCG motif were filtered from the rest of the data. It was observed that OA G20 showed differences in the methylation for 0 µM-Cu and 30 µM-Cu biofilm samples. Methylation levels above 75% were detected at 341 and 424 genomic positions in the 0 µM and 30 µM-Cu biofilm samples, respectively. Figure 4 shows genomic positions of methylated TCCG motifs for both control and 30 µM-Cu biofilm samples containing genes with methylation > 75%.

To further determine the differences in DNA methylation patterns in the two experimental conditions, the search was narrowed to common methylated genomic positions. A total of 1418 common methylated positions were identified for both conditions, followed by mapping them to their respective gene identifiers (Supplementary File S5). Table 2 presents the list of the top 20 genes that were significantly methylated (> 85%) in CB and CuB samples, and play a key role in transport, chemotaxis, motility and quorum sensing. To explore the detailed function of methylated genes, functional annotation and pathway enrichment analyses were performed using GO (UniProtKB) and KEGG databases, respectively.

**Table 2:** Top 20 methylated genes in biofilms exposed to 30 µM-Cu ion concentrations and control (0 µM-Cu).

Gene ID	Gene description	Percent modified_0 µM	Gene ID	Gene description	Percent modified_30 µM
Dde_0259	ABC-type transporter, integral membrane subunit	1	Dde_0205	SEC-C motif domain protein	1
Dde_0830	Polysaccharide export protein, PEP-CTERM system-associated	1	Dde_0215	Glycosyl transferase family 2	1
Dde_1563	Acriflavin resistance protein	0.95	Dde_0243	Peptidase M23	1
Dde_2681	Uncharacterized protein	0.95	Dde_0310	Cl-channel voltage-gated family protein	1
Dde_1151	Metal dependent phosphohydrolase	0.94	Dde_0759	Putative ABC transporter solute-binding protein	1
Dde_0729	Uncharacterized protein	0.93	Dde_1138	5-carboxymethyl-2-hydroxymuconate Delta-isomerase (EC 5.3.3.10)	1
Dde_0378	Flagellar biosynthetic protein FliR	0.9	Dde_1190	Cobalamin (Vitamin B12) biosynthesis CbiM protein	1
Dde_0703	Methyl-accepting chemotaxis sensory transducer with Pas/Pac sensor	0.9	Dde_0225	AsmA family protein	0.95
Dde_0729	Uncharacterized protein	0.9	Dde_0048	UspA domain-containing protein	0.94
Dde_0411	ABC3 transporter permease protein domain-containing protein	0.89	Dde_2547	Uncharacterized protein	0.94
Dde_1327	ABC-type transporter, integral membrane subunit	0.89	Dde_0544	histidine kinase (EC 2.7.13.3)	0.93
Dde_1602	Xenobiotic-transporting ATPase (EC 3.6.3.44)	0.89	Dde_1151	Metal dependent phosphohydrolase	0.93
Dde_2704	Adenosylcobinamide-GDP ribazoletransferase (EC 2.7.8.26) (Cobalamin synthase) (Cobalamin-5’-phosphate synthase)	0.89	Dde_1193	Component of nickel ABC transport system	0.93
Dde_3023	4Fe-4 S ferredoxin iron-sulfur binding domain-containing protein	0.89	Dde_2093	DNA ligase (EC 6.5.1.2) (Polydeoxyribonucleotide synthase [NAD(+)])	0.93
Dde_0173	Ribonuclease II	0.88	Dde_2138	Cytochrome-c3 hydrogenase (EC 1.12.2.1)	0.93
Dde_0265	4Fe-4 S ferredoxin iron-sulfur binding domain-containing protein	0.88	Dde_2562	Pyridine nucleotide-disulfide oxidoreductase family protein	0.93
Dde_0417	Acetylornithine transaminase (EC 2.6.1.11)	0.88	Dde_2615	Aspartyl/glutamyl-tRNA(Asn/Gln) amidotransferase subunit B (Asp/Glu-ADT subunit B) (EC 6.3.5.-)	0.93
Dde_0770	Uncharacterized protein	0.88	Dde_2679	HPt domain-containing protein	0.93
Dde_1165	histidine kinase (EC 2.7.13.3)	0.88	Dde_2766	Glycosyl transferase group 1	0.93
Dde_1181	Oligopeptide/dipeptide ABC transporter, ATPase subunit (EC 3.6.3.24)	0.88	Dde_0703	Methyl-accepting chemotaxis sensory transducer with Pas/Pac sensor	0.92

### Functional annotation and pathway enrichment analyses

**Fig. 5:**
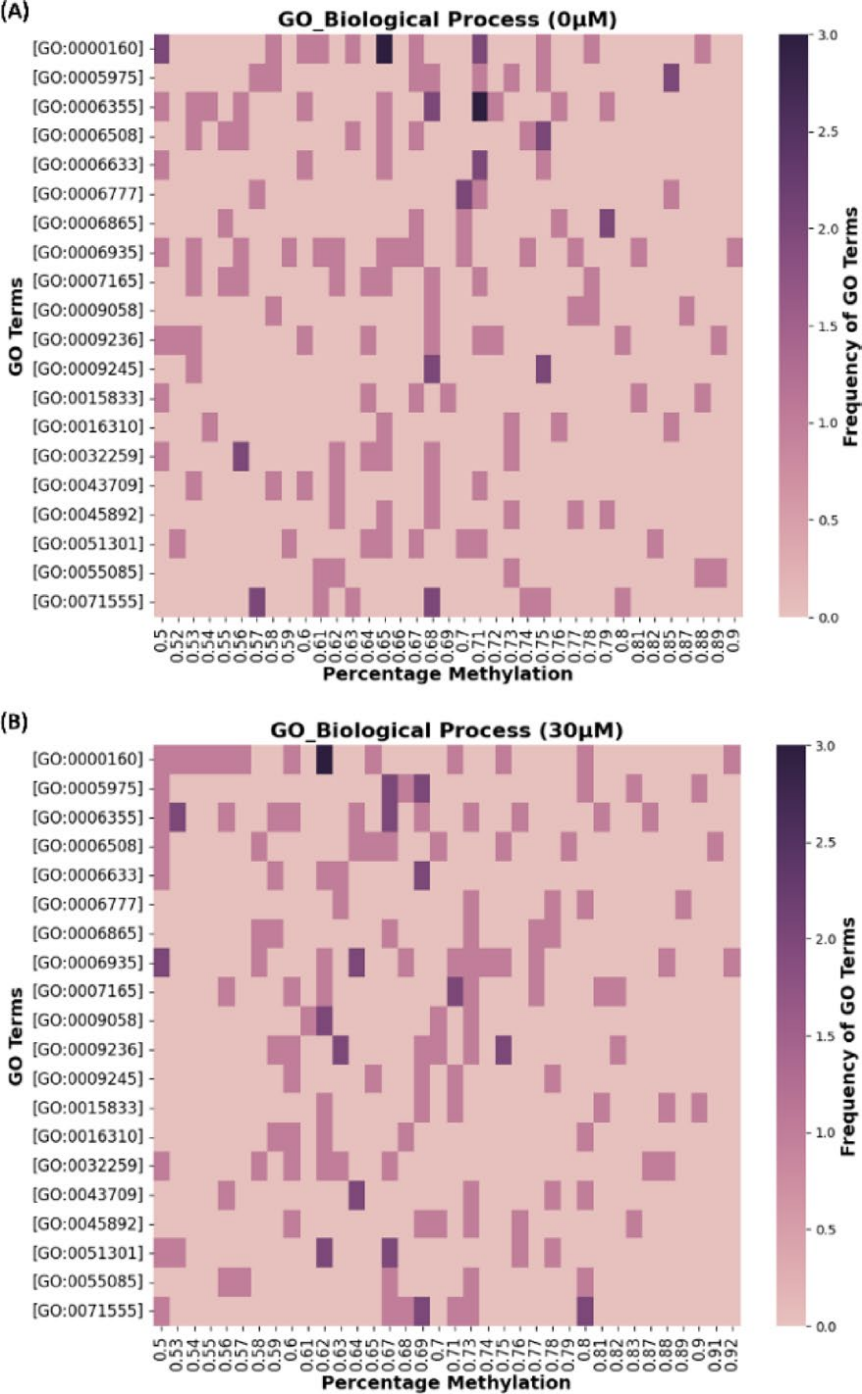
Biological process gene ontology enrichment analysis of differentially methylated genes. (**A**) For control biofilm and (**B**) For biofilms exposed to 30 µM-Cu ions. The heatmap depicts the 20 most recurring GO_BP terms across both experimental conditions, highlighting differences in methylation patterns between two conditions.

GO functional analysis was performed for methylated genes present in both control and test (30 µM-Cu) samples. GO analysis is a standard approach that has been utilized to understand and infer biological and molecular mechanisms from experimental data^60,61^. GO terms are broadly categorized into three separate GO terms, such as biological process (BP), molecular function (MF), and cellular component (CC)^36,62^. Our analyses revealed that the shared methylated genes were involved in multiple BPs and MFs (Supplementary File S6). The top 20 enriched functional terms are presented in Fig. 5; this section entails a detailed explanation of only the top 3 GO terms for BP and MF categories shared by both the experimental conditions.

For the GO_BP, category the most enriched functional terms in the descending order were involved in phosphorelay signal transduction (GO:0000160, 15 methylated genes), transcription regulation (GO:0006355, 14 methylated genes), and chemotaxis (GO:0006935, 14 methylated genes) (Fig. 5). The methylated genes included in GO:0000160 take part in the bacterial signal transduction system. Phosphorelay is an intricate variant of the two-component system (TCS), allowing the bacteria to sense and respond to changing environmental conditions by activating or suppressing the gene expression^63,64^.

**Fig. 6:**
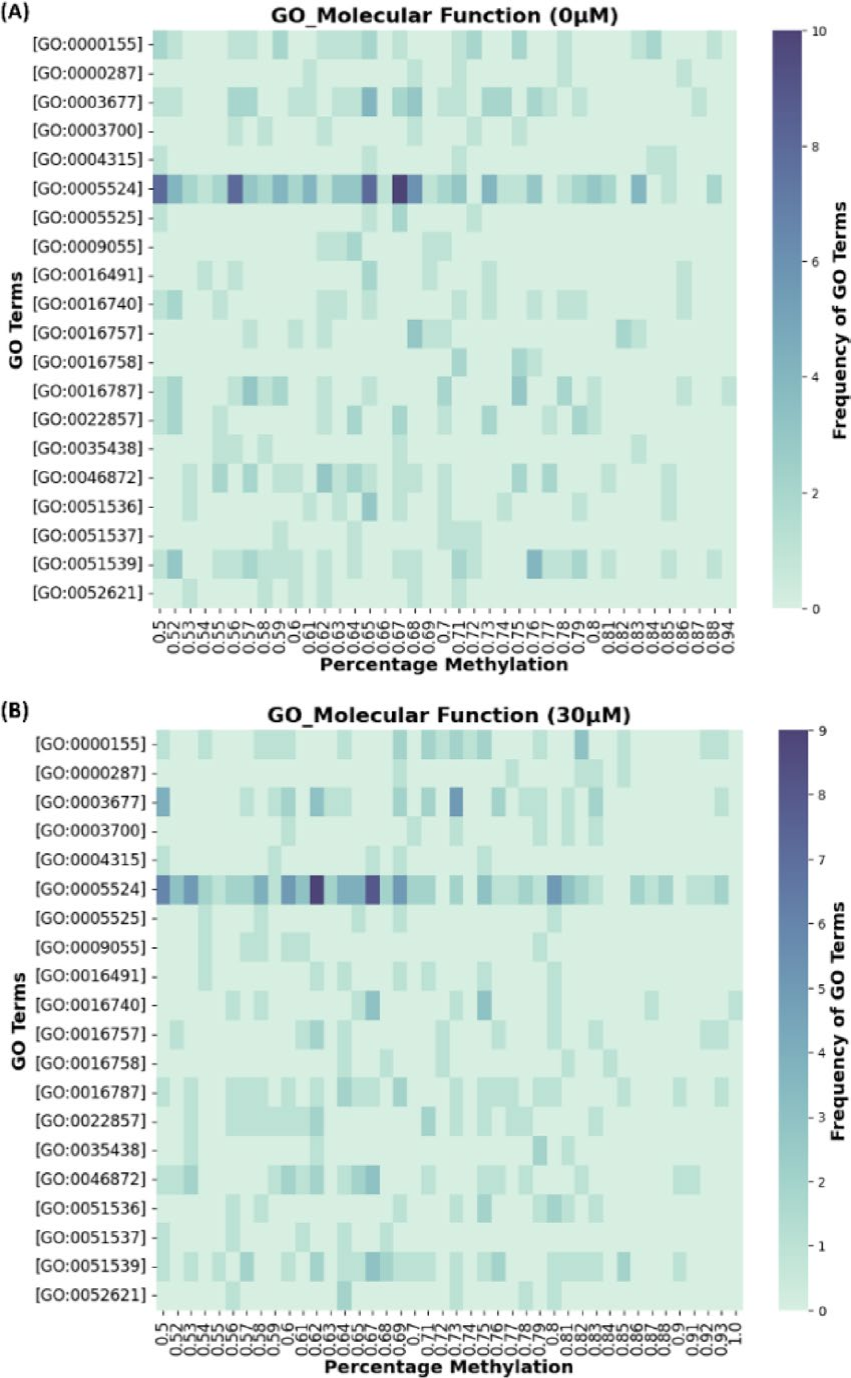
Molecular function gene ontology functional analysis of differentially methylated genes. (**A**) For control biofilm and (**B**) For biofilms exposed to 30 µM-Cu ions. The heatmap depicts the 20 most recurring GO_BP terms across both experimental conditions, highlighting differences in methylation patterns between them.

Previous studies have elaborated on the role of phosphorelay signal transduction in regulating *E. coli* and *Serratia marcescens* biofilm formation and stress response^65,66^. The enrichment of GO:0006935 suggests the active reprogramming of gene regulation which is often coupled with changes in DNA methylation patterns^67^. For example, DNA methylation regulates the expression of genes involved in phase variation and phenotypic heterogeneity^68^. In *E. coli* DNA methylation has also been reported to prevent the binding of oxidative response-regulator protecting the cells against oxidative stress^5^. Additionally, the genes associated with GO:0006935 play a role in chemotaxis, a process that allows bacteria to move towards favorable conditions^69^. The role of chemotaxis in metal-stress response and biofilm formation has previously been explored^70,71^. Moreover, the relationship between DNA methylation and chemotaxis has been studied in various microorganisms like *E. coli*, *Salmonella enterica*, and *Thermotoga maritima*^72–74^. These studies reported that the methylation/demethylation cycle of methyl-accepting chemotaxis protein allowed the bacteria to detect and respond to chemical changes^75^. In MF category, ATP binding (GO:0005524, 102 methylated genes), DNA binding (GO:0003677, 30 methylated genes), and iron-sulfur (Fe-S) cluster binding (GO:0051539, 29 methylated genes) functional terms were enriched (Fig. 6). The highest number of methylated genes belongs to ATP binding, implying ATP-dependent processes like expression of efflux pumps, signal transduction system, biofilm formation by kinase-driven pathways, and other cellular processes are actively being modulated by the bacteria to survive under stress^76–78^. The Fe-S cluster binding genes have also been reported to confer resistance to metal stress by upregulating genes encoding Fe-S cluster proteins^79^. Also, for the CC category, plasma membrane (GO:0005886, 105 methylated genes), membrane (GO:0016020, 104 methylated genes), and cytosol (GO:0005829, 81 methylated genes) GO terms demonstrated significant enrichment (Supplementary Figure S2 and File S6).

**Fig. 7:**
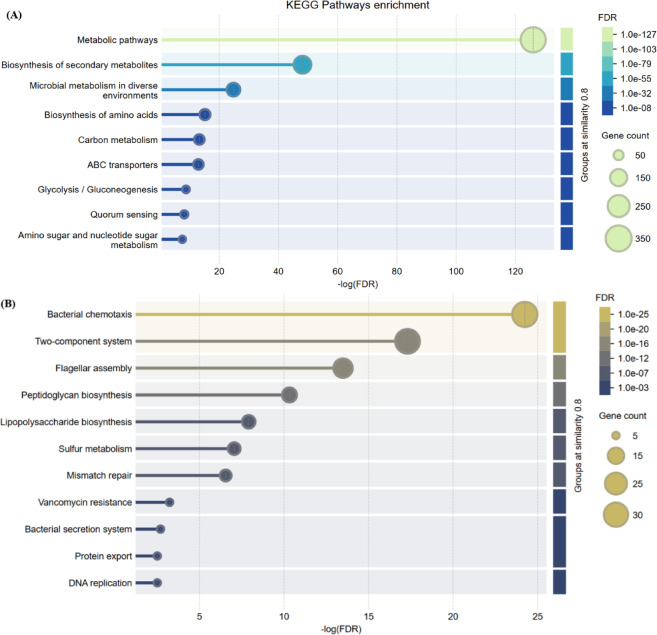
STRING-DB pathway enrichment analysis. Most enriched pathways for genes exhibiting differential methylation patterns in both control and 30 µM-Cu biofilm samples. (**A**) and (**B**) correspond to critical pathways in OA G20 survival and stress response. Statistical significance is displayed as -log(FDR) on the x-axis, where FDR is the False Discovery Rate; larger values indicate more significant pathway enrichment^80^.

Next, KEGG pathway analysis (Supplementary File S7) was performed to understand the functional roles of the methylated genes. The KEGG functional annotation revealed that the methylated genes were involved in a total of 112 unique pathways. Pathway enrichment analysis, conducted using STRING-DB, revealed that the most significantly enriched pathways were associated with microbial metabolism in diverse environments, ABC transporters, two-component systems, chemotaxis, sulfur metabolism, flagellar assembly, etc. (Fig. 7)^80^. The role of crucial pathways related to stress response and biofilm formation are discussed in detail in the following sections.

## Discussion

DNA methylation can regulate transcription by modulating the accessibility of promoter and transcription binding factors. For example, the Pyelonephritis-associated pilus (pap) operon in *E. coli* provides evidence that methylation state of GATC motifs can control the phase-variation of pilus gene whereas the methylation of GATC sites in the promoter region of antigen 43 (*agn43*) gene leads to transcription initiation^9^. In *Caulobacter*, the methylation of GANTC in the promoter region of *CcrM* regulates the expression of *ctrA* (global response regulator). The methylation state of these sites affects binding of the transcriptional activator *ctrA* and thereby influences *ccrM* expression^5^. Additionally, methylation also creates epigenetic ON/OFF states, whereby switching in methyltransferase activity leads to coordinated regulation of multiple genes, generating phenotypic heterogeneity in traits including virulence, motility, and biofilm formation. Such mechanisms of formation of diverse DNA methylation patterns which further control gene expression are complex and are explained in detail elsewhere^6,81^. DNA methylation has also been reported in bacterial stress adaptation mechanisms, for example, Gurbanov et al. postulated that alterations in methylation status of metal-acclimated *Gordonia sp.* (Cd: 49% increase; Pb: 52% decrease; Ag: 42% decrease) potentially facilitates stress adaptation by changing DNA-protein interactions thereby modulating activation or repression the genes^82^. Our earlier research on OA G20 demonstrated an upregulation of DNMT-encoding genes when exposed to elevated copper concentrations of 15 µM compared to control (0 µM). This enhanced methyltransferase activity at high Cu levels suggests that DNA methylation likely modifies gene expression as an adaptive response to metal stress^48^. The role of methylation in regulating other critical pathways has been discussed in detail in the following sections.

### DNA methylation in carbon metabolism genes

Metal ion stress can disrupt the central carbon and energy metabolism in bacteria. Bacteria utilize several strategies to overcome stress, which is mostly done by regulating the gene expression. Under cadmium stress, *Streptococcus pneumoniae* cells showed perturbations in key cellular processes, like carbon metabolism, glycolysis, fatty acid biosynthesis and metal homeostasis^83^. Additionally, a study on *Desulfovibrio* sp. revealed that during biofilm formation, genes related to carbon flow and energy conversion showed a shift in gene expression relative to the planktonic cells^84^. This regulation of gene expression suggests that adjustments in key cellular processes like carbon and energy metabolism are stress-response strategies adopted by bacteria^85^.

In our current study, differential methylation patterns (DMP) were observed for genes involved in carbon and energy metabolism where a total of 57 genes were found to be differentially methylated (Supplementary File S8). For 30 µM-Cu samples, genes Dde_1796 (PM_CB = 0.62, PM_CuB = 0.75), Dde_3597 (PM_CB = 0.50, PM_CuB = 0.62), and Dde_3489 (PM_CB = 0.58, PM_CuB = 0.62) showed increased methylation compared to control and were associated with gluconeogenesis. Conversely, most genes from the pentose phosphate pathway (PPP), like Dde_3470 (PM_CB = 0.75, PM_CuB = 0.62), Dde_3502 (PM_CB = 0.62, PM_CuB = 0.62), and Dde_2631 (PM_CB = 0.70, PM_CuB = 0.53) were either more methylated or equally methylated in the CB samples relative to the CuB. A potential reason for this transition from higher methylation of CuB genes in gluconeogenesis/glycolysis to decreased methylation in the pentose phosphate pathway (PPP) may be driven by the cell’s need to regulate redox balance and generate NADPH for Fe-S cluster stabilization, suggesting a metabolic adaptation mechanism to maintain cellular homeostasis^83,86,87^.

Previous understanding of SRB’s response under stress emphasized the regulation of energy metabolism and iron homeostasis^85,88,89^. However, through multi-omics studies, it has been established that modulation of other pathways like signal transduction, cell motility, chemotaxis, biofilm formation and efflux pumps also play a crucial role in bacterial survival under stress^35,48,88,90,91^.

### Influence of DNA methylation in stress response pathways

#### Methylation of amino-acid synthesis genes

Cu toxicity in bacteria is mainly due to its ability to catalyze the production of reactive oxygen species (ROS), inducing oxidative stress^92^. Bacteria alleviate this stress through the synthesis of glutathione, which is a low-molecular-weight thiol compound derived from three amino acids such as glutamine, cysteine, and glycine^93^. Glutathione has been previously studied for its role in multiple processes like Cu-stress resistance^93,94^, acid-induced stress defense^95^, metal-homeostasis^96,97^ and protection against ROS^98^. Methylation analysis revealed differential patterns among glutamine biosynthesis genes (glutamate synthase (Dde_1250), acetylglutamate kinase (Dde_2015), glutamine synthetase (Dde_0102), glutamine synthetase, type I (Dde_0104)), whereas cysteine biosynthesis genes (homocysteine S-methyltransferase (Dde_2328), cystathionine beta-lyase (Dde_0276)) were moderately methylated, with significant methylation variability in glycine hydroxymethyltransferase (Dde_2432, PM_CB = 0.77, PM_CuB = 0.59), involved in glycine synthesis (Supplementary File S9).

The methylome analysis also detected methylation in other amino acid biosynthesis genes, including genes coding for diaminopimelate decarboxylase (Dde_1798, PM_CB = 0.62, PM_CuB = 0.75 and Dde_2664, PM_CB = 0.74, PM_CuB = 0.92) involved in the production of lysine. Whereas the arginine biosynthesis gene *argJ* was significantly methylated in CuB samples (PM = 0.82) relative to the control (PM = 0.52).

Arginine and lysine metabolism have been studied in *Salmonella*,* B. subtilis* and *E. coli* for their role in cellular protection against oxidative and acid stress^99–101^. In addition, valine, leucine, isoleucine, and arginine metabolic pathways were also found to be methylated in our study (Supplementary File S9), the role of these amino acids has been associated with biofilm modulation in *Pseudomonas* sp. in anaerobic environments^102,103^. The differential methylation patterns in all these genes are suggestive of an intricate signaling circuit that exists between amino acid biosynthesis and cellular mechanisms like biofilm formation and metal stress response. However, the link between methylation patterns and the regulation of amino acid biosynthesis genes requires future investigation.

#### Methylation changes in efflux pumps

The predominant means by which bacteria confer heavy metal resistance is by using efflux pumps. The main categories of efflux pumps present in the microorganisms include ABC transporters, Multidrug-efflux systems, and Resistance Nodulation Cell Division (RND) transporters^104^. The efflux pumps protect bacteria against heavy metal stress by reducing intracellular metal ion concentration or by exporting the metal ion outside from the cell^105,106^. The analysis of CB and CuB indicated that genes associated with (a) organic ion transport, (b) phosphate and amino acid transporters, (c) oligosaccharide, monosaccharide and lipid transporters, (d) peptide transporter, and (e) metal ion transporters were methylated. The ABC transporter genes Dde_0232 (*tupC*), Dde_0233 (*tupB*), and Dde_3309 (*tupA*) linked with tungsten (W) transport were moderately methylated with no changes in methylation patterns between the two experimental conditions. The molybdenum (Mo) ABC transporters *modA* (Dde_0155), *modB* (Dde_3519), and *modF* (Dde_1055) exhibited differential methylation patterns, with Dde_1055 showing the highest methylation levels (PM_CB = 0.6, PM_CuB = 0.85). Previous research has demonstrated that regulation of W and Mo transporters is essential for cellular metallostasis, and various redox reactions involved in carbon and sulfur metabolism^107,108^. It has also been reported that *ModA* is essential for anaerobic growth and virulence in *Pseudomonas aeruginosa*^109^.

Comparative methylation analysis revealed distinct patterns across *cbiM* (Dde_1190), cbiN/L (Dde_11910), *cbiO* (Dde_1193), *znuA*, and *znuC* under two experimental conditions. The Co/Ni ABC transporter gene Dde_1190 exhibited the highest changes in DNA methylation (PM_CB = 0.74, PM_CuB = 1), while Dde_2209 (PM_CB = 0.58, PM_CuB = 0.67) was moderately methylated. Also, the zinc transporter gene Dde_2209 (*znuC*, PM_CB = 0.64, PM_CuB = 0.86) showed notable differences in methylation patterns. Other ABC transporters genes that showed methylation were zinc (*znuA*), biotin (*bioYN*), glutamine (*glnH*,* glnP*), and heme (*ccmA*,* ccmB*) (Supplementary File S10). Albeit the role of efflux pumps has been widely studied for metal resistance, an increasing number of studies show their involvement in biofilm formation and QS^110^. For example, iron, zinc, amino acids, and inorganic ion transporters of *Moraxella catarrhalis* promote cell adhesion^111,112^. Whereas heme transporters of *M. tuberculosis* and *H. influenzae* contribute to the structure of adhesion proteins^112–114^. In addition, amino acid and oligopeptide transporters are required for cell survival and regulation of gene expression involved in cell adhesion, thereby promoting biofilm formation^112^. Even though gene expression studies are required to understand the influence of DNA methylation of the above-mentioned genes, the differences in DNA methylation in CB and CuB samples are indicative of a potential regulatory mechanism adapted by OA G20 to cope with metal stress.

### Differential methylation patterns in signal transduction systems

The signal transduction system is responsible for controlling cellular processes in prokaryotes. The primary mode of signal transduction identified in bacteria is two-component regulatory systems (TCS), which allows bacteria to sense and respond to environmental changes^115^. The name ‘two-component’ indicates the presence of two distinct components; a receptor histidine kinase which senses the stimulus and a response regulator (RR) which regulates the cellular response^116^. TCS has been studied for its role in controlling gene expression, biofilm formation, and cell motility by employing several cross-regulation mechanisms in response to a wide variety of stimuli^117,118^. Among these are osmoregulation (OmpR family), nitrogen regulation (NtrC family), bacterial chemotaxis (Che), citrate regulation (CitB family), and quorum sensing^119^. Our data analysis identified that genes related to these families were methylated with PM ≥ 0.5. The role of these genes in OA G20’s biofilm formation and stress response has been discussed in the sections below.

#### Chemotaxis

The bacterial chemotaxis system is the most intricate of all the TCS pathways which regulate cell motility in response to environmental stimuli^115^. The significance of chemotaxis system in biofilm formation remains ambiguous. For example, P. Merritt et al. concluded that chemotaxis is required for *Agrobacterium tumefaciens* for biofilm development^120^. While, in *E. coli*, biofilm formation proceeds independently of chemotactic function^121^. Our KEGG pathway analysis revealed that a total of 33 genes involved in bacterial chemotaxis, including genes coding for methyl-accepting chemotaxis proteins (MCPs), *cheA*, *cheB*, *cheW*, *cheR*, *cheV*, and *cheX* were methylated in both the experimental conditions (Supplementary File S11). Comparative methylome analysis showed the most significant differential PM occurred in response regulator *cheW* (Dde_3100, PM_CB = 0.56, PM_CuB = 0.92) followed by methyltransferase *cheR* (Dde_1198, PM_CB = 0.56, PM_CuB = 0.88), suggesting that methylation may play a central role in fine-tuning chemotactic responses under Cu stress. Details of other methylated genes involved in chemotaxis are listed in Supplementary File S11.

MCPs initiate the bacterial chemotactic response by detection of stimulus, followed by signal transmission to a histidine kinase, *cheA* through *cheW*^122^. The resulting modulation of *cheA* activity influences *cheY* phosphorylation, which determines its motor-binding capability. The protein *cheY* can also be dephosphorylated by one or more phosphatases, including *cheC*, *cheX*, and *cheZ*^123,124^. Other response regulators like *cheB* and *cheR* further control the propagation of signals through MCPs by removing or adding the methyl group to the receptor, respectively^125^. Finally, *cheV*, a combination of *cheW* and *cheY*), influences the regulation of MCPs signaling. Previous studies have shown that chemotaxis signaling pathways ultimately control two forms of bacterial motility: flagellar-based or type IV pili-dependent movement^124,126^. Additionally, our previous study on OA G20 under Cu stress also revealed a co-regulation of genes involved in flagellar and chemotaxis assembly^48^.

#### Flagellar and motility-related genes

In response to environmental stimuli, bacteria mediate motility by employing two structures; pili and flagella^127^. Bacteria exhibit various motility mechanisms, including flagella-driven swimming in liquid media^128^ or Type IV pili-driven twitching motility along the surfaces^129^. The effect of cell motility on biofilm formation has been previously explored in various microorganisms, including *P. aeruginosa*^130,131^, *E. coli*^132^, *Desulfovibrio* sp.^133^, and *S. marcescens*^134^. For example, *E. coli* mutants with defective flagellar biosynthesis (*fla*^-^) and motility (*mot*^-^) demonstrated defects in surface attachment and biofilm formation^121^. Also, studies on *P. aeruginosa* revealed that both flagellar motility and Type-IV pili are required for biofilm formation^135,136^.

The structural organization of periplasmic flagella in Gram-negative bacteria consist of four principal components: the flagellar motor, hook, filament, and export apparatus. The flagellar motor is further comprising rotor and stator assemblies. The rotor assembly incorporates several components: the membrane/supramembrane (MS) ring functioning as the basal platform, the cytoplasmic (C) ring mediating flagellar rotational directionality, the peptidoglycan (P) and lipopolysaccharide (L) rings providing structural integrity to the rotating rod, and the rod component facilitating mechanical transmission. A coordinated expression of more than 60 genes is required to achieve functional flagellar assembly^137–139^. Examination of our results showed that genes encoding critical flagellar components: the C-ring protein (*fliN*), structural ring proteins (*flgH*), stator complex proteins (*motA* and *motB*) mediating C-ring rotation^139,140^, rod complex proteins (*flgB*, *flgC*, and *flgG*), export apparatus components (*flhA*, *fliQ*, and *fliR*) and ATPase complex proteins (*fliH* and *fliI*) orchestrating temporal and spatial assembly^141,142^, and filament proteins (*fliC* and *fliD*) were methylated in both experimental conditions (Supplementary File S11). In addition to the flagellar genes, our analysis revealed that genes encoding Type IV pilus assembly *pilZ* exhibited significant differential methylation patterns. Examples of some genes with difference in methylation include Dde_0821(PM_CB = 0.58, PM_CuB = 0.81), Dde_1029 (PM_CB = 0.67, PM_CuB = 0.79), Dde_0203 (PM_CB = 0.83, PM_CuB = 0.79) (Supplementary File S11). Prior research has shown that DNA methylation acts as an ON/OFF switch in genes which control various functions like cell motility and biofilm formation^143^.

#### Quorum sensing

Bacterial quorum sensing (QS) can be defined as cell-to-cell communication conspires through the production and detection of chemical signals like autoinducers (AI) enabling bacteria to regulate gene expression in response to variations in cell density^144^. Bacteria rely on QS to regulate various cellular processes important for adaptation to changing environmental conditions, this includes biofilm formation, synthesis of antimicrobial compounds, and pathogenesis^118,145^. In Gram-negative bacteria, cell communication is achieved through producing AHLs (N-acyl homoserine lactones), a class of AIs^145,146^. The role of QS in conferring metal stress tolerance and biofilm formation in bacteria such as *Pseudomonas aeruginosa* has also been demonstrated^144,147^. Differential methylation patterns were observed in our methylome dataset for the LuxR family genes *dctP* (Dde_2216, PM_CB = 0.61, PM_CuB = 0.80) and *dctQ* (Dde_1274, PM_CB = 0.52, PM_CuB = 0.56) (Supplementary File S11). These LuxR-type quorum sensing regulatory proteins exhibit dual signal recognition capability, responding to both exogenous AHLs and non-AHL signaling molecules^148^. Zhang et al. previously demonstrated that inactivation of the *dctP* gene in *Vibrio alginolyticus* resulted in reduced cellular motility and biofilm development capabilities^149^.

#### Methylation of *NtrC* family genes

Various transcription regulators and sigma factors control the regulatory network governing bacterial biofilm formation^150,151^. *RpoN*, an alternative sigma factor, promotes biofilm formation by initiating transcription through interactions with bacterial enhancer-binding proteins (bEBPs)^150^. One such group of these bEBPs belong to the nitrogen regulatory protein C (NtrC) family, which is reported to regulate nitrogen assimilation, production of exopolysaccharides, and biofilm formation in bacteria^152,153^. In our data, we observed that genes related to *glnA* (Dde_0102, PM_CB = 0.64, PM_CuB = 0.88), responsible for glutamine synthesis; *glnB* (Dde_2310, PM_CB = 0.56, PM_CuB = 0.67), a key regulator in nitrogen assimilation; *glnD* (Dde_2309, PM_CB = 0.52, PM_CuB = 0.56), acts a nitrogen sensor in bacteria; *ntrY* (Dde_3000, PM_CB = 0.52, PM_CuB = 0.54), multi-sensor signal transduction kinase; *rpoN* (Dde_1711, PM_CB = 0.50, PM_CuB = 0.50) and other sigma-54 factors were methylated (Supplementary File S11). Glutamine synthetase is encoded by gene *glnA* (Dde_0102, PM_CB = 0.64, PM_CuB = 0.88), which is required for the synthesis of glutamine, one of the most important amino acids used by bacteria for producing essential metabolites and nitrogen-containing compounds^154^. In *Bacillus subtilis*, glutamine plays a critical role in biofilm formation and cell proliferation around biofilm colony edges^154,155^. Loss of the *gltA* and *gltB* genes, responsible for glutamate production, inhibited *B. subtilis* biofilm formation capabilities^156,157^. *NtrY*, traditionally known for nitrogen metabolism, has also been reported to regulate cell motility and EPS biosynthesis^158^. These methylation patterns suggest a potential regulatory mechanism that could further modulate gene expression, however, their further experimental validation using RT-PCR or transcriptomics is required to understand the implications of DNA methylation on gene expression.

### Gene methylation patterns associated with biofilm formation

OA G20 uses biofilm formation as a key strategy to protect itself from metal ion stress^47^. It has been previously reported that DNA methylation plays a role in the regulation of biofilm formation in various bacteria like (i) *Salmonella enterica*, where DNA adenine methyltransferase (Dam) modulates the length of O-anitgen chain of Lipopolysaccharide (LPS) by either directly regulating Wzzst production or influencing RcsB and PmrA protein expression, which subsequently alters LPS structure and affects biofilm development^7,8^; (ii) *Acinetobacter baumannii*, in which two DNA methylation-related genes showed biofilm-specific expression, being present only in sessile cells while remaining completely silent in all planktonic growth phases^159^. (iii) *Clostridium difficile*, the 6mA-MTase CD2758 catalyzes methylation of adenine residues within the conserved 5’-CAAAAA-3’ recognition sequence. Transcriptomic analysis via RNA-seq revealed that methylation at these target sites modulates the expression of genes encoding proteins critical for biofilm formation and cell adhesion^160^. Based on the KEGG pathway and GO analysis, several genes playing key roles in biofilm formation and EPS biosynthesis in OA G20 were identified as methylated, with some genes showing significant differences in methylation between the two experimental conditions. Genes related to lipopolysaccharide, peptidoglycan, bacterial secretion system, EPS biosynthesis, cell shape, adhesion, and division were among the pathways that are related to biofilm formation, as described in the sections below.

#### Lipopolysaccharide biosynthesis

In Gram-negative bacteria, like *E. coli*, *Salmonella*, and *Desulfovibrio*, the major components of the biofilm matrix are colonic acid, exopolysaccharide, protein, carbohydrates, and extracellular DNA^161^. All these components play a significant role in maintaining the cell-to-cell connections and biofilm structure^162^. Lipopolysaccharide (LPS) is a predominant component of the outer membrane of the cell envelope, functions as a primary defense mechanism against environmental stresses and plays a critical role in initial cell attachment and biofilm formation, and a barrier to toxic metal ions^163,164^. Our KEGG analysis revealed a total of 14 genes were differentially methylated in CB and CuB samples (Supplementary File S12). Some of the genes with significant differences in methylation patterns were hydrolase, Acyl–UDP-N-acetylglucosamine O-acyltransferase (Dde_1374, PM_CB = 0.75, PM_CuB = 0.65); 3-deoxy-D-manno-octulosonate cytidylyltransferase (CMP-KDO) (Dde_3687, PM_CB = 0.75, PM_CuB = 0.62), and D-glycero-D-manno-heptose-7-phosphate 1-kinase (HBP) (Dde_0028, PM_CB = 0.74, PM_CuB = 0.62), where Kdo and HBP form the inner core of LPS in *E. coli* and *Salmonella*^161^. Regulation of genes involved in LPS biosynthesis significantly affects the viability of the biofilms^165^. This is demonstrated by the essential role of CMP-KDO in incorporating KDO into LPS structure, as mutants deficient in KDO within their LPS exhibited reduced biofilm viability^166,167^. On the contrary, *Shigella flexneri* mutants lacking HBP showed an increase in biofilm formation and cell adhesion^168^. While the regulatory mechanisms of DNA methylation on LPS remain poorly understood, extensive immunological studies have documented the impact of LPS on DNA methylation patterns in eukaryotic systems^5,169–171^.

#### Peptidoglycan biosynthesis

Peptidoglycan (PG), maintains shape and provides structural support against cytoplasmic turgor pressure, is a fundamental component of the bacterial cell envelope^172^. In Gram-negative bacteria, its structure is characterized by linear glycan chains composed of alternating N-acetylglucosamine (GlcNAc) and N-acetylmuramic acid (MurNAc) residues^172,173^. L, D-transpeptidases (LDTs) are essential enzymes that catalyze the final polymerization reaction during peptidoglycan cross-linking^174^. The role of LDT in biofilm formation has been studied in *P. aeruginosa*, where deletion of the cross-linking gene *Ldt*_*pae1*_ resulted in decreased biofilm formation^173^. The analysis of our methylome data revealed that a total of 13 genes including, Bifunctional transpeptidase/transglycosylase (Dde_0269, PM_CB = 0.74, PM_CuB = 0.64); UDP-N-acetylenolpyruvoylglucosamine reductase (Dde_1044, PM_CB = 0.71, PM_CuB = 0.61); phospho-N-acetylmuramoyl-pentapeptide-transferase (Dde_1039, PM_CB = 0.68, PM_CuB = 0.53); and UDP-N-acetylglucosamine–N-acetylmuramyl pentapetide (MurG)(Dde_1042,PM_CB = 0.68, PM_CuB = 0.67) had a differential methylation pattern (Supplementary File S12). There is no direct evidence of the influence of DNA methylation on PG synthesis. However, DNA adenine methylase mutants of *S. enterica* exhibited unstable cell envelopes, resulting from improper binding of periplasmic protein (Tol) and peptidoglycan-associated lipoprotein (PAL) to PG^175^.

#### Cell division and shape

DNA methylation has been studied in bacteria to control cell division and bacterial phenotypic variations^7,17,21,22^. For example, in *Caulobacter crescentus*, cell cycle-regulated DNMTs CcrM methylates adenine residues of GATC and GNTC sites. In mutants lacking *ccrM*, viability and cell division defects were observed which were compensated by increased expression of cell division gene *ftsZ*. Moreover, the authors reported that the *ΔccrM* cells were more elongated than the wild type, suggesting DNA methylation regulates the expression of genes involved in variation in phenotype^176^. Additionally, cell division and shape play crucial roles in biofilm architecture, influencing the development and structure of microbial communities^177^. After the attachment of bacteria on a surface, microcolonies are formed by the process of cell division^178^ and adapts to changes in environmental conditions by altering their cellular morphology^179^. Previous studies on *E. coli*^180^ and *M. tuberculosis*^181^ showed that there was an increase in the cell size when bacteria were exposed to stress. Similarly, studies in OA G20 and Mn-oxidizing bacteria reported cell elongation when exposed to metal ion stress^47,48,182^.

Our GO analysis of the methylome data observed genes involved in cell division and cell shape exhibited differences in their methylation, including cell division protein *ftsX* (Dde_0115, PM_CB = 0.82, PM_CuB = 0.78 ), cell shape-determining protein *mreB* (Dde_0996, PM_CB = 0.50, PM_CuB = 0.60), and cell shape-determining protein *mreC* (Dde_0995, PM_CB = 0.82, PM_CuB = 0.50). Studies on *Fusobacterium nucleatum*, a Gram-negative anaerobic bacterium have shown that *ftsX* plays a crucial role in biofilm formation, where *ftsX* mutants formed weak adherent biofilms^183^. The actin homolog MreB coordinates with transmembrane protein MreC to mediate bacterial cell elongation^184^. The role of *mreB* and *mreC* has been previously explored for their role in altering cellular morphology and biofilm development^185^. For example, the deletion of *mreB* in *B. subtilis* resulted in spherical cell morphology, ultimately causing cell death through lysis, suggesting *mreB* is important for maintaining cell shape^186^. In our data, the gene *mreC* observed a significant difference in methylation in CB and CuB samples. Previous studies in *P. aeruginosa* showed that mutation in the *mreC* gene increased the biofilm tolerance to gentamicin^187^. Similarly, the DNA methylated-driven regulation of *mreB* has also been reported by several authors^188–190^.

Other methylated genes that are responsible for dictating bacterial cell shape, elongation and division are genes related to PG synthesis, such as *ftsW*, *murA*, *murB*, *murC*, *murI*, *murJ*, and *mraY*^191^. The description of these genes along with their methylation percentages is presented in the Supplementary File S12. Our previous study showed that OA G20 cells elongated under Cu stress compared to the control. Since DNA methylation is known to regulate genes involved in cell division and shape (like *ftsX* and *mreB*), these variations in phenotypes suggest that additional genes controlling cell division or cell elongation might be regulated by DNA methylation in OA G20, either directly or indirectly.

#### Cell adhesion and polysaccharide synthesis

Bacterial biofilms are encapsulated with a self-produced extracellular polymer matrix (ECM), composed of polysaccharides (PS), eDNA, lipids, carbohydrates and proteins^192^. PS play a crucial role in biofilm formation by aiding surface adhesion, microbial interactions, and maintaining biofilm structure. Additionally, bacteria can modify PS production as an adaptive strategy to protect the cells from stressful conditions^193^. Several genes involved in PS biosynthesis and cell adhesion were differentially methylated in our current study. The significantly methylated genes include genes encoding polysaccharide export protein (Dde_0830, PM_CB = 1, PM_CuB = 0.82), polysaccharide biosynthesis protein *capD* (Dde_0360, PM_CB = 0.54, PM_CuB = 0.70 ), exopolysaccharide biosynthesis polyprenyl glycosylphosphotransferase (Dde_0829, PM_CB = 0.61, PM_CuB = 0.71 ), polysaccharide pyruvyl transferase (Dde_0419, PM_CB = 0.52, PM_CuB = 0.67), serine O-acetyltransferase (Dde_3081, PM_CB = 0.72, PM_CuB = 0.54), and various genes coding for diguanylate cyclase (like, Dde_1069, PM_CB = 0.60, PM_CuB = 0.78; Dde_0077, PM_CB = 0.68, PM_CuB = 0.80) (Supplementary File S12).

Our previous study demonstrated that when OA G20 biofilms were grown in 30 µM-Cu conditions, qPCR analysis revealed the polysaccharide synthesis gene (*poI*) expression was significantly upregulated with a log2 fold = 1.77 compared to control biofilm conditions^47^, suggesting that DNA methylation might have a positive effect on the regulation of gene expression. On the contrary, Rekha Nair et al.^194^ reported that DNA methylation negatively regulated the expression of the EPS production gene, *epsR* in *Ralstonia pseudosolanacearum*, which could be attributed to the methylation of the *epsR* promoter region. Similar results were reported in *Xanthomonas axonopodis*, where overexpression of putative DNMTs, EadM resulted in decreased tolerance to ciprofloxacin and EPS synthesis but increased resistance to desiccation stress^195^. These findings demonstrate a link between DNA methylation and EPS synthesis.

### Perspectives and highlights

DNA methylation patterns in OA G20 biofilms were investigated using ONT sequencing, comparing samples exposed to 30 µM-Cu ion stress with control. Several methylation motifs were identified in both conditions that couldn’t be linked to known R-M systems, suggesting the presence of orphan DNMTs. This highlights how DNA methylation in SRB remains largely unexplored. The study focused on the TCCG motif, which showed methylation in both CB and CuB conditions. While the experiment was originally conducted in duplicate, technical limitations of the analysis pipeline prevented the processing of biological replicates. Additionally, the MicrobeMod pipeline lacked in-built tools for statistical and differential analysis between samples. Although the experiment was originally conducted in biological duplicates, technical limitations of the analysis pipeline prevented the processing of biological replicates. The MicrobeMod pipeline is specifically designed for single-sample analysis and lacks the capability to handle multiple replicates simultaneously or perform integrated statistical comparisons between biological replicates. These constraints necessitated a single-sample analytical approach for each condition. Future studies would benefit from employing workflows designed to analyze biological replicates and perform statistical analyses.

Our investigation revealed differences in DNA methylation patterns between CuB and CB samples. These findings align with growth condition-dependent methylation variations that were previously observed in *E. coli*^10^. The CuB showed increased methylation sites compared to CB samples, suggesting that Cu stress influences methylation patterns throughout the OA G20 genome. This finding expands our understanding of bacterial DNA methylation beyond its traditional role in defending against foreign DNA, pointing to a more complex adaptive mechanism. While we have not established a direct link between DNA methylation and metal stress tolerance in OA G20, studies in other bacteria like *E. coli* and *V. cholerae* have shown that DNA methylation can regulate oxidative stress and cell envelope stress responses, respectively^5^.

While eukaryotic DNA methylation and its impact on gene expression and cellular phenotypes have been well-documented^196,197^. Understanding these mechanisms in bacteria is still emerging. Based on our findings discussed in the sections above, we propose that DNA methylation in OA G20 may function as a regulator of gene expression, potentially influencing key cellular processes such as biofilm formation, extracellular polysaccharide synthesis, cell morphology, stress responses, lipopolysaccharide and peptidoglycan synthesis, and two-component systems including quorum sensing and chemotaxis. However, since our study did not incorporate gene expression analyses, we cannot determine if Cu stress affects DNA methylation or if DNA methylation alters Cu stress response in OA G20.

## Conclusion

This study delivers the first in-depth epigenomic characterization of a SRB, laying the groundwork for future research into the epigenetic regulation and adaptive strategies employed by SRB under environmental stress conditions. The main outcomes observed in our current research include: (a) No DNMTs in the REBASE database have been experimentally confirmed to target motifs identified in this study; (b) CB showed methylation in three motifs like TCCG, CCCGCCCG, and CGGGAT, where TCCG motif was most methylated with base modifications in 78,022 genomic positions; (c) In CuB samples, TCCG and GCANCTGCGS motifs were found to be methylated with 63,315 genomic positions in TCCG motifs; and (d) Differential methylation patterns were observed in genes involved in critical cellular process like stress response, amino-acid and lipopolysaccharide biosynthesis, signal transduction system etc. While previous studies have demonstrated that DNA methylation independently can influences biofilm formation and stress adaptation, the integrated mechanisms linking these processes remain poorly understood. This study provides initial evidence for changes in DNA methylation patterns when OA G20 biofilms are exposed to metal ion stress, yet the functional consequences require further investigation. Future research employing transcriptomics and proteomics approaches will be essential to validate the proposed methylation-dependent regulatory mechanisms and establish mechanistic connections between copper stress, epigenetic modifications, and biofilm regulation in OA G20.

## Supplementary Information

Below is the link to the electronic supplementary material.

Supplementary Material 1

Supplementary Material 2

Supplementary Material 3

Supplementary Material 4

Supplementary Material 5

Supplementary Material 6

Supplementary Material 7

Supplementary Material 8

Supplementary Material 9

Supplementary Material 10

Supplementary Material 11

Supplementary Material 12

Supplementary Material 13

## Data Availability

The sequence data files (.BAM) were submitted to the NCBI under Bioproject ID PRJNA1240245.

## Code availability

The used codes are given in the Supplementary Materials.
