# Supplementary material for: Long-read methylome analysis of Oleidesulfovibrio alaskensis G20 biofilm under copper stress
Source: Sci Rep. 2025 Oct 31;15:38250. doi: 10.1038/s41598-025-22029-8 (PMC12579266; doi:10.1038/s41598-025-22029-8)
Supplement: Supplementary file 1 — Supplementary Material 1 [file 41598_2025_22029_MOESM1_ESM.docx]

**File S1: R-scripts and codes for circus plot and heat maps**

**R-script for Circos plot (Figure 3):**

///////

library(circlize)

library(readxl)

library(tidyr)

circos.clear()

# Load and process the first dataset (bc03_output.xlsx)

data1 <- readxl::read_excel('bc03_output.xlsx')

data1$`gene start` <- sapply(strsplit(as.character(data1$`gene start`), ";"), `[`, 1)

data1 <- data1[, c(1, 2, 3, 12)]

data1 <- separate(data1, `gene start`, into = c("col1", "col2"), sep = ",")

colnames(data1) <- c('position', 'locus_tag', 'start', 'end', 'percent_modified')

data1 <- na.omit(data1)

# Define genome length and convert position to Mb for the first dataset

genome_length <- as.numeric(3153905) # Assuming genome length

genome_length_mb <- genome_length / 1e6

data1$position_mb <- as.numeric(data1$position) / 1e6

# Load and process the second dataset (bc01_output.xlsx)

data2 <- readxl::read_excel('bc01_output.xlsx')

data2 <- data2[, c(1, 2, 4, 5, 14)]

colnames(data2) <- c('position', 'locus_tag', 'start', 'end', 'percent_modified')

data2 <- na.omit(data2)

# Convert position to Mb for the second dataset

data2$position_mb <- as.numeric(data2$position) / 1e6

# Load and process the new dataset with descriptions

new_data <- readxl::read_excel('Cicos_plot_labelled genes.xlsx')

colnames(new_data) <- c('position', 'locus_tag', 'description', 'percent_modified')

# Convert position to Mb for the new dataset

new_data$position_mb <- as.numeric(new_data$position) / 1e6

# Prepare data in BED format (start and end positions are the same for point labels)

bed <- data.frame(

chr = "Genome", # Single sector for the genome

start = new_data$position_mb,

end = new_data$position_mb,

label = new_data$locus_tag

)

# Increase overall plot size

pdf("circos_plot.pdf", width = 10, height = 10) # Increase resolution, width, and height

# Adjust margin parameters

circos.par(

cell.padding = c(0, 0, 0, 0),

track.margin = c(0.01, 0.01)

)

circos.par("canvas.xlim" = c(-1.2, 1.2), "canvas.ylim" = c(-1.2, 1.2))

# Initialize circos with a single sector for the whole genome

circos.clear() # Clear any previous plots

circos.initialize(

factors = "Genome", # Single factor for the entire genome

xlim = c(0, genome_length_mb) # Genome range in Mb

)

# Add genomic labels for descriptions outside the outer circle

circos.genomicLabels(

bed,

labels.column = 4, # Column with descriptions (labels)

side = "outside",# Place labels outside the circle

niceFacing = TRUE,

cex = 1, # Make labels smaller

col = "darkgreen", # Color of the labels

line_col = "gray" # Color of lines connecting labels to positions

)

# Add an outer track to represent the genome in Mb

circos.trackPlotRegion(

ylim = c(0, 1), # Define y-axis limits for the track

track.height = 0.1, # Increase the track height

panel.fun = function(x, y) {

xlim <- get.cell.meta.data("xlim")

circos.axis(

h = "top",

major.at = seq(xlim[1], xlim[2], by = genome_length_mb / 10), # Divide into ticks

labels = round(seq(xlim[1], xlim[2], by = genome_length_mb / 10), digits = 1),

labels.cex = 0.5, # Make axis labels smaller

labels.facing = "clockwise"

)

}

)

# Add black lines to represent methylation positions in Mb scale (outer circle)

circos.segments(

sector.index = "Genome", # Use "Genome" as the single sector index

x0 = data1$position_mb, # Start of each line (in Mb)

y0 = rep(0.5, nrow(data1)), # Fixed y-coordinate for all lines (middle of track)

x1 = data1$position_mb, # End of each line (same as start for vertical lines)

y1 = rep(1, nrow(data1)), # Extend to top of track

col = "black",

lwd = 1

)

# Filter data for methylation percentages above a threshold (outer circle)

filtered_data_outer <- data1[data1$percent_modified > 0.75, ]

# Add bars for methylation percentages above threshold (outer circle)

circos.trackPlotRegion(

ylim = c(0, 100), # Y-axis range for percentages (0% to 100%)

track.height = 0.18,

panel.fun = function(x, y) {

bar_width <- 0.00005 # Reduce bar width (smaller than before)

for (i in seq_len(nrow(filtered_data_outer))) {

circos.rect(

xleft = filtered_data_outer$position_mb[i] - bar_width, # Narrower bar width

xright = filtered_data_outer$position_mb[i] + bar_width,

ybottom = 0,

ytop = filtered_data_outer$percent_modified[i] * 100, # Scale to percentage

col = "orange",

border = "red"

)

}

circos.axis(

h = "left",

labels.cex = 0.4,

major.at = seq(0, 100, by = 20), # Tick marks from 0% to 100%

labels.facing = "inside"

)

}

)

# Filter data for methylation percentages above a threshold (inner circle)

filtered_data_inner <- data2[data2$percent_modified > 0.75, ]

# Add an inner circle track for the second dataset

circos.trackPlotRegion(

ylim = c(0, 100), # Y-axis range for percentages (0% to 100%)

track.height = 0.18, # Adjusted track height

panel.fun = function(x, y) {

bar_width <- 0.00005 # Bar width for inner circle

for (i in seq_len(nrow(filtered_data_inner))) {

circos.rect(

xleft = filtered_data_inner$position_mb[i] - bar_width,

xright = filtered_data_inner$position_mb[i] + bar_width,

ybottom = 0,

ytop = filtered_data_inner$percent_modified[i] * 100, # Scale to percentage

col = "blue",

border = "darkblue"

)

}

circos.axis(

h = "left", # Axis on the left side of the track

labels.cex = 0.4, # Font size for axis labels

major.at = seq(0, 100, by = 20), # Tick marks from 0% to 100%

labels.facing = "inside" # Labels facing inside

)

}

)

legend(

x = "topright", # Position on the bottom-right side

inset = c(0, 0.1), # Adjust horizontal and vertical position (negative moves outside)

legend = c("Total Methylated Regions", "30 µM (>75%)", "0 µM (>75%)"), # Updated labels with micro symbol (µ)

col = c("black", "red", "blue"), # Colors corresponding to each label

lwd = c(1, NA, NA), # Line width for black lines (outer circle)

pch = c(NA, 15, 15), # Symbols for 30 µM and 0 µM bars

pt.cex = 1.5, # Symbol size

bty = "n", # No border around legend box

cex = 0.8, # Font size for legend text

xpd = TRUE, # Allow drawing outside the plot area

y.intersp = 1.5 # Increase vertical spacing between legend items (adjust as needed)

)

dev.off()

////////////

**Code for Heat Maps (Figure 4 and 5):**

//////////

import pandas as pd

df = pd.read_excel('/content/GO_plot_30vs0.xlsx', sheet_name=1)

freq_XYZ_A = df['GO_ID_BP'].value_counts().reset_index()

freq_XYZ_B = df['GO_ID_MF'].value_counts().reset_index()

freq_XYZ_C = df['GO_ID_CC'].value_counts().reset_index()

freq_XYZ_A.columns = ['Value', 'Freq_BP']

freq_XYZ_B.columns = ['Value', 'Freq_MF']

freq_XYZ_C.columns = ['Value', 'Freq_CC']

combined_freq = freq_XYZ_A.merge(freq_XYZ_B, on='Value', how='outer').merge(freq_XYZ_C, on='Value', how='outer').fillna(0)

with pd.ExcelWriter('/content/top_20_terms_30µM.xlsx') as writer:

    freq_XYZ_A.head(20).to_excel(writer, sheet_name='Top 20 GO_ID_BP', index=False)

    freq_XYZ_B.head(20).to_excel(writer, sheet_name='Top 20 GO_ID_MF', index=False)

    freq_XYZ_C.head(20).to_excel(writer, sheet_name='Top 20 GO_ID_CC', index=False)

print("\nTop 20 Frequencies for GO_ID_BP:", freq_XYZ_A.head(20))

print("\nTop 20 Frequencies for GO_ID_MF:", freq_XYZ_B.head(20))

print("\nTop 20 Frequencies for GO_ID_CC:", freq_XYZ_C.head(20))

///////////////

import pandas as pd

import seaborn as sns

import matplotlib.pyplot as plt

from matplotlib.colors import LinearSegmentedColormap

df = pd.read_excel('/content/GO_plot_30vs0.xlsx', sheet_name=0)

top_20 = df['GO_ID_MF'].value_counts().nlargest(20).index

filtered_df = df[df['GO_ID_MF'].isin(top_20)]

pivot_table = filtered_df.pivot_table(index='GO_ID_MF', columns='percent methylation_0µM_bc01_MF', aggfunc='size', fill_value=0)

blue_cubehelix = sns.cubehelix_palette(start=0.5, rot=-0.5, light=0.9, dark=0.3, as_cmap=True)

plt.figure(figsize=(10, 8))

ax=sns.heatmap(pivot_table, cmap=blue_cubehelix, annot=False)

plt.title('GO_Molecular Function (0µM)', fontsize=16, fontweight='bold')

plt.xlabel('Percentage Methylation', fontsize=14, fontweight='bold')

plt.ylabel('GO Terms', fontsize=14, fontweight='bold')

ax.set_xticklabels(ax.get_xticklabels(), fontsize=12)

ax.set_yticklabels(ax.get_yticklabels(), fontsize=12)

colorbar = ax.collections[0].colorbar

colorbar.set_label('Frequency of GO Terms', fontsize=14, fontweight= 'bold')

plt.show()

///////
