# Supplementary material for: Long-read methylome analysis of Oleidesulfovibrio alaskensis G20 biofilm under copper stress
Source: Sci Rep. 2025 Oct 31;15:38250. doi: 10.1038/s41598-025-22029-8 (PMC12579266; doi:10.1038/s41598-025-22029-8)
Supplement: Supplementary file 10 — Supplementary Material 10 [file 41598_2025_22029_MOESM10_ESM.pdf]

Figure S1: Graphs for read alignment of CB and CuB

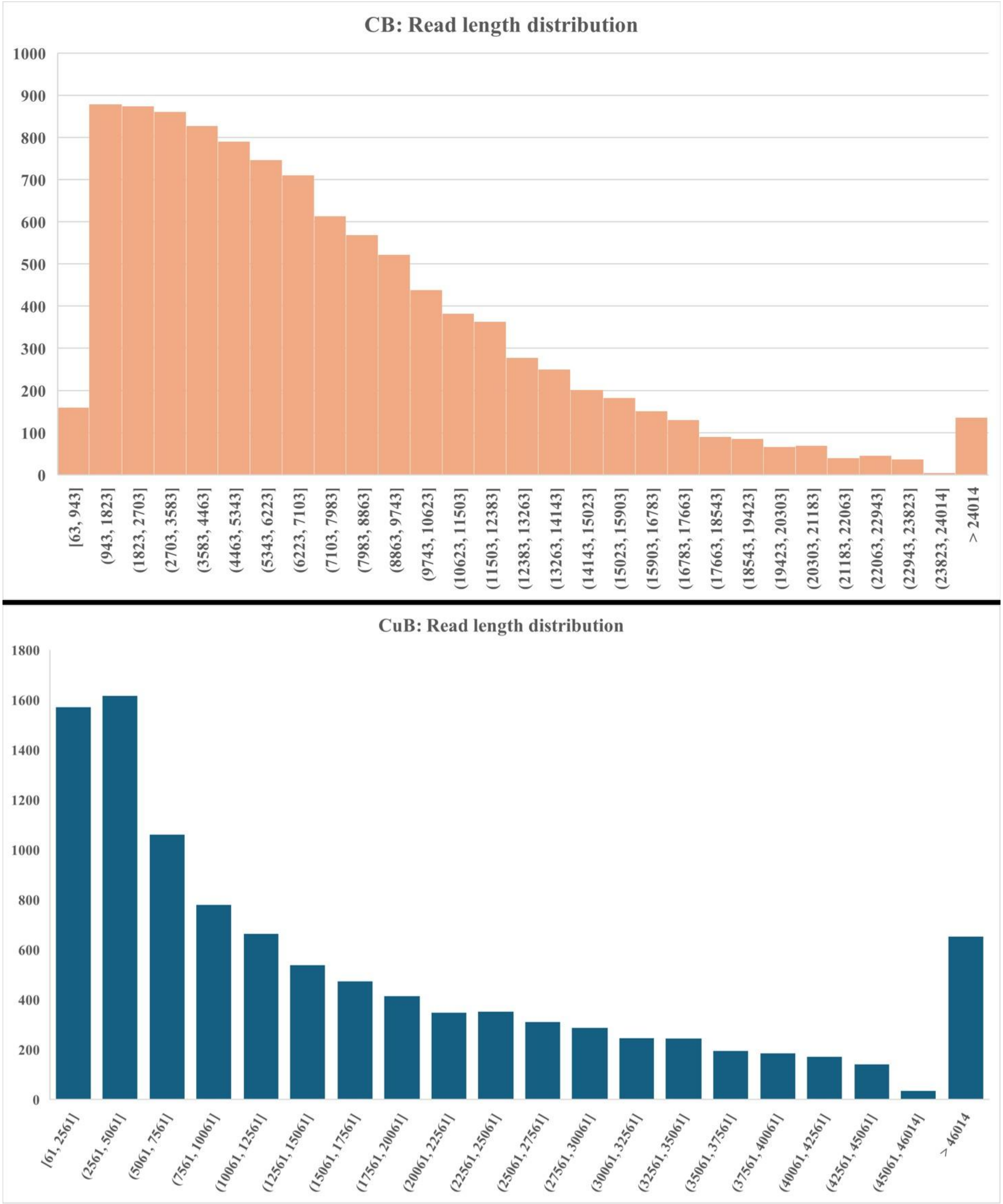

Figure S2: Heatmap depicting GO_CC functional analysis for both 0 µM and 30 µM.

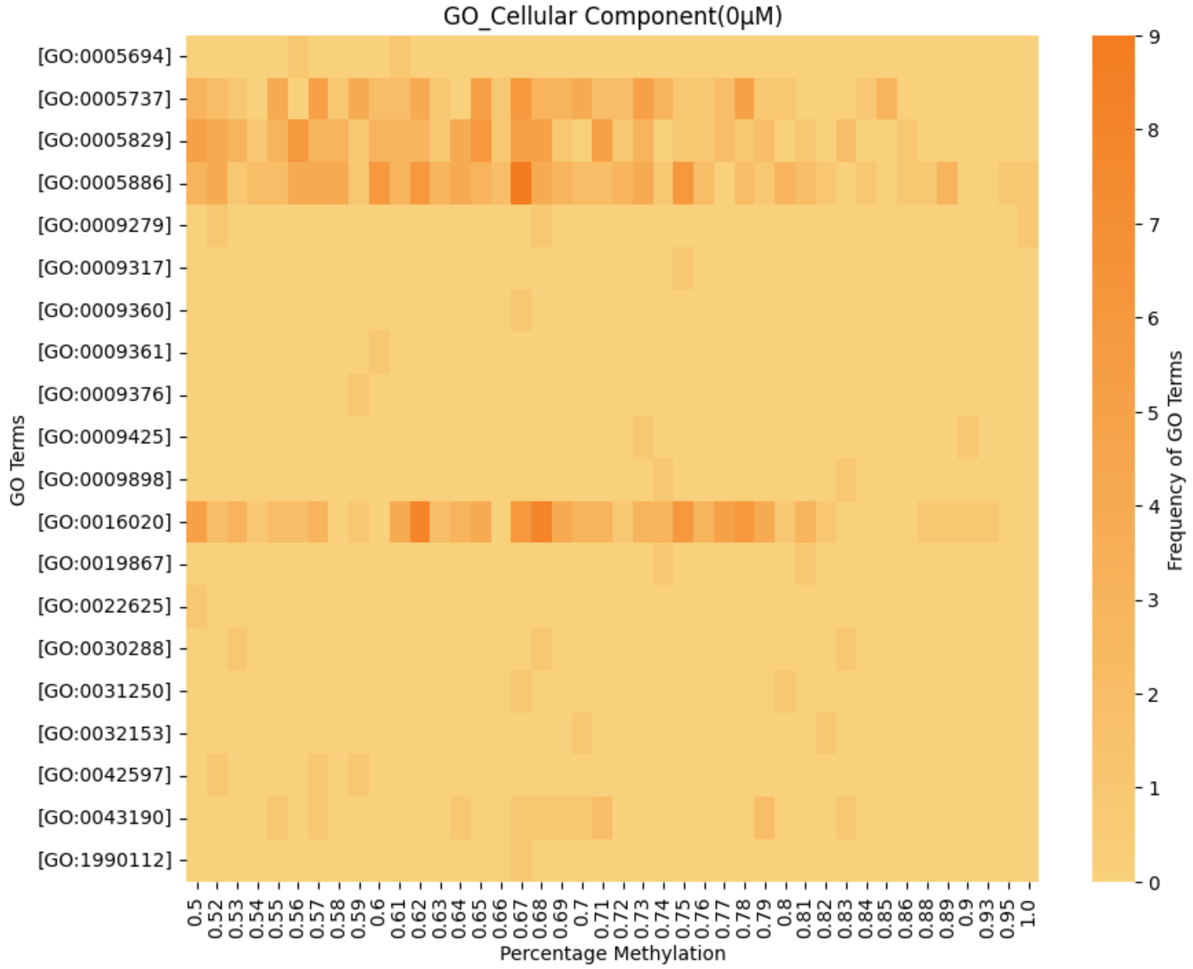

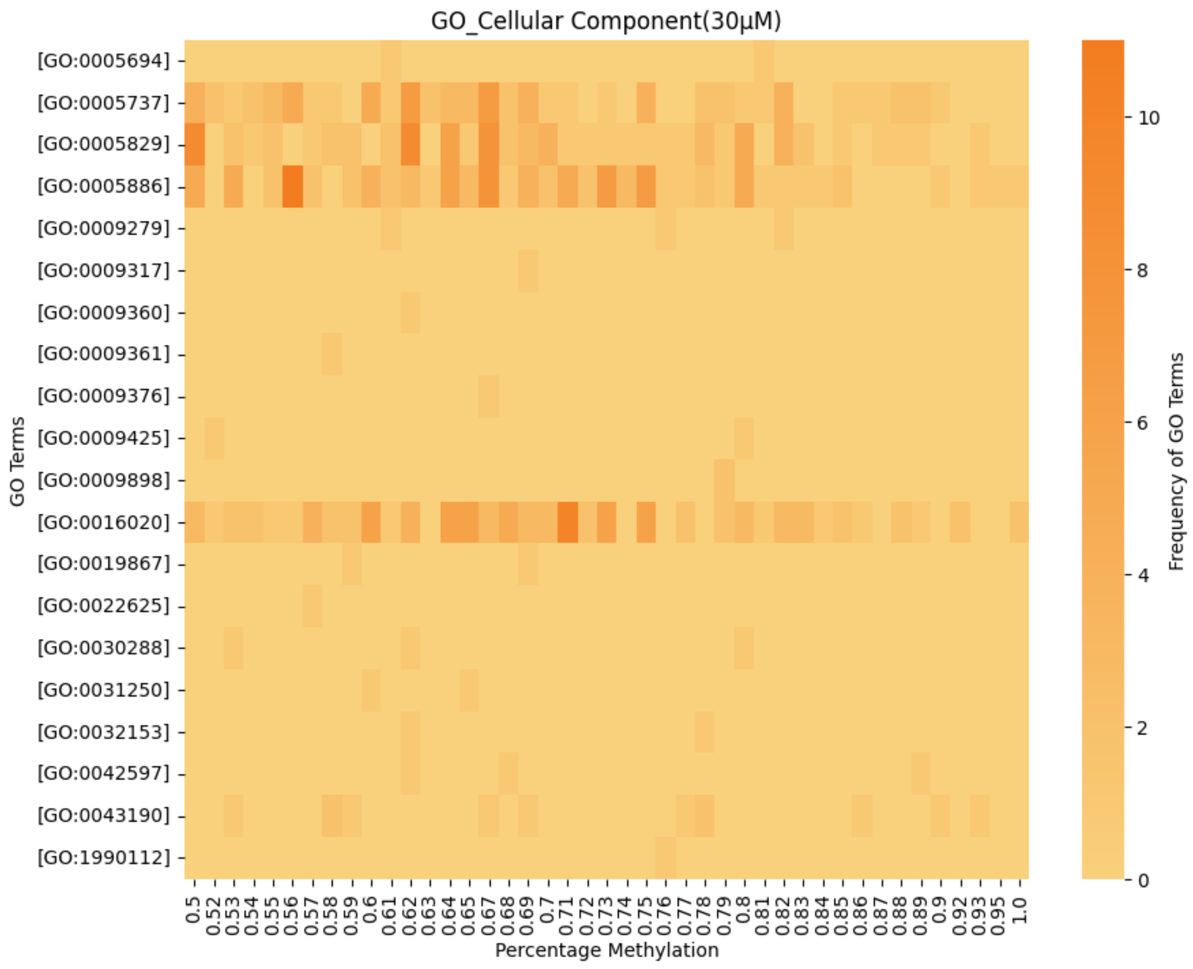

GO_Cellular Component(30μM)
GO Terms
[GO:0005694]
[GO:0005737]
[GO:0005829]
[GO:0005886]
[GO:0009279]
[GO:0009317]
[GO:0009360]
[GO:0009361]
[GO:0009376]
[GO:0009425]
[GO:0009898]
[GO:0016020]
[GO:0019867]
[GO:0022625]
[GO:0030288]
[GO:0031250]
[GO:0032153]
[GO:0042597]
[GO:0043190]
[GO:1990112]
0.5 0.52 0.53 0.54 0.55 0.56 0.57 0.58 0.59 0.6 0.61 0.62 0.63 0.64 0.65 0.67 0.68 0.69 0.7 0.71 0.72 0.73 0.74 0.75 0.76 0.77 0.78 0.79 0.8 0.81 0.82 0.83 0.84 0.85 0.86 0.87 0.88 0.89 0.9 0.92 0.93 0.95 1.0
Percentage Methylation
Frequency of GO Terms
0
2
4
6
8
10
